# Synaptic Vesicle Precursors and Lysosomes Are Transported by Different Mechanisms in the Axon of Mammalian Neurons

**DOI:** 10.1016/j.celrep.2020.107775

**Published:** 2020-06-16

**Authors:** Raffaella De Pace, Dylan J. Britt, Jeffrey Mercurio, Arianne M. Foster, Lucas Djavaherian, Victoria Hoffmann, Daniel Abebe, Juan S. Bonifacino

**Affiliations:** 1 Neurosciences and Cellular and Structural Biology Division, Eunice Kennedy Shriver National Institute of Child Health and Human Development, National Institutes of Health, Bethesda, MD 20892, USA; 2 Division of Veterinary Resources, National Institutes of Health, Bethesda, MD 20892, USA; 3 Lead Contact

## Abstract

BORC is a multisubunit complex previously shown to promote coupling of mammalian lysosomes and *C. elegans* synaptic vesicle (SV) precursors (SVPs) to kinesins for anterograde transport of these organelles along microtubule tracks. We attempted to meld these observations into a unified model for axonal transport in mammalian neurons by testing two alternative hypotheses: (1) that SV and lysosomal proteins are co-transported within a single type of “lysosome-related vesicle” and (2) that SVPs and lysosomes are distinct organelles, but both depend on BORC for axonal transport. Analyses of various types of neurons from wild-type rats and mice, as well as from BORC-deficient mice, show that neither hypothesis is correct. We find that SVPs and lysosomes are transported separately, but only lysosomes depend on BORC for axonal transport in these neurons. These findings demonstrate that SVPs and lysosomes are distinct organelles that rely on different machineries for axonal transport in mammalian neurons.

## In Brief

De Pace et al. show that lysosomes and synaptic vesicle precursors (SVPs) are distinct organelles that move separately from the soma to the axon in rat and mouse neurons. Moreover, they demonstrate that the BLOC-1-related complex (BORC) is required for the transport of lysosomes but not SVPs in mouse neurons.

## Graphical Abstract

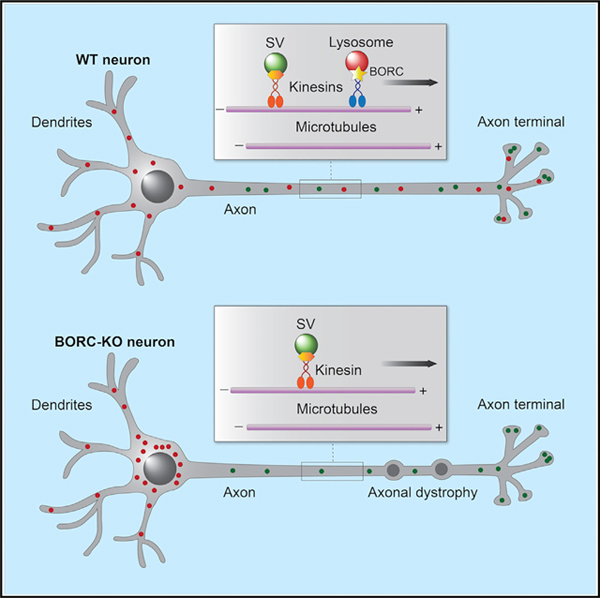

## INTRODUCTION

The ability of organelles to move within the cytoplasm is critical for the distribution of their activities to different intracellular locations (van Bergeijk et al., 2016). Nowhere is this ability more important than in neurons, which have a highly asymmetric organization with extremely long processes such as the axon and dendrites. The axon, in particular, has uniformly polarized bundles of microtubules that serve as highways for long-distance transport of organelles in both anterograde (outward) and retrograde (inward) directions (Maday et al., 2014). Anterograde and retrograde movements are mediated by coupling of the organelles to kinesin and dynein-dynactin microtubule motors, respectively. In most cases, this coupling is not direct but depends on various adaptor or scaffold proteins and their regulators. The critical role of axonal organelle movement in neuronal physiology is underscored by the existence of numerous neuro-developmental and neurodegenerative disorders caused by mutations in components of the organelle transport machinery (Zhao et al., 2001; Reid et al., 2002; Puls et al., 2003; Erlich et al., 2011; Rivière et al., 2011; Poirier et al., 2013; Esmaeeli Nieh et al., 2015; Melo et al., 2015; Charng et al., 2016; Duis et al., 2016; Nicolas et al., 2018).

Two organelles that have been well documented to undergo long-range axonal transport are lysosomes and synaptic vesicle precursors (SVPs). Lysosomes are membrane-bound, acidic organelles that are primarily involved in intracellular degradation of biomacromolecules but also participate in various non-degradative processes such as nutrient and growth factor signaling and plasma membrane repair (Ballabio and Bonifacino, 2020). Axonal lysosomes are particularly heterogeneous with regard to their degradative capacity and acidity (Lee et al., 2011; Gowrishankar et al., 2015; Farías et al., 2017; Cheng et al., 2018; Farfel-Becker et al., 2019), a property that may reflect the existence of populations of lysosomes with distinct functions or maturational states (Ferguson, 2019). In mammals, anterograde lysosome transport depends on coupling to several members of the kinesin family, especially the kinesin-1 proteins KIF5A, KIF5B, and KIF5C, and kinesin-3 proteins KIF1A and KIF1Bβ (Nakata and Hirokawa, 1995; Tanaka et al., 1998; Matsushita et al., 2004; Guardia et al., 2016; Farías et al., 2017). KIF5A, KIF5C, and KIF1A are brain specific, whereas KIF5B and KIF1Bβ are ubiquitous (Okada et al., 1995; Xia et al., 1998; Kanai et al., 2000). Coupling of lysosomes to these kinesins is mediated by the BLOC-1-related complex (BORC), which is composed of eight subunits named BLOS1 (also known as BORCS1 and BLOC1S1), BLOS2 (BORCS2/BLOC1S2), snapin (BORCS3/BLOC1S7), KXD1 (BORCS4), myrlysin (LOH12CR1/BORCS5), lyspersin (C17orf59/BORCS6), diaskedin (C10orf32/BORCS7), and MEF2BNB (BORCS8) (Pu et al., 2015). BORC associates with the cytosolic leaflet of the lysosomal membrane in part through a myristoyl group at the N terminus of the myrlysin subunit (Pu et al., 2015). BORC then promotes recruitment of the small GTPase ARL8 (which in mammals exists as two paralogs, ARL8A and ARL8B) (Pu et al., 2015), probably by acting as a ARL8 guanine nucleotide exchange factor (GEF) (Niwa et al., 2017). ARL8 subsequently binds KIF5A/KIF5B/KIF5C indirectly via the adaptor protein SKIP (Nakata and Hirokawa, 1995; Rosa-Ferreira and Munro, 2011) and the kinesin light chain (KLC) or KIF1A by direct interaction with the CC3 domain of this protein (Niwa et al., 2016). In neurons, kinesin-1 proteins appear particularly important for transport of lysosomes in the axon (Farías et al., 2017), although a role for kinesin-3 proteins in this process has not been ruled out.

SVPs are still poorly characterized organelles that arise in the neuronal soma and move along the axon, carrying synaptic vesicle (SV) proteins to pre-synaptic terminals (Goldstein et al., 2008). The mechanisms of SVP transport have been studied in most detail in the nematode *C. elegans*. Remarkably, kinesin coupling to SVPs in this organism is also mediated by BORC and ARL8 (Klassen et al., 2010; Wu et al., 2013; Zheng et al., 2014; Niwa et al., 2016, 2017). However, in this case, the relevant kinesin is UNC-104 (Niwa et al., 2016), the nematode ortholog of the mammalian kinesin-3 proteins KIF1A and KIF1Bβ, which are also involved in SVP transport in mammals (Okada et al., 1995; Nakamura et al., 2002; Guedes-Dias et al., 2019).

The observations that BORC and ARL8 mediate axonal transport of both lysosomes in mammals and SVPs in *C. elegans* raise two alternative possibilities: (1) that axonal lysosomes and SVPs are one and the same organelle and (2) that axonal lysosomes and SVPs are distinct organelles but use the same machinery for coupling to kinesins. In support of the first possibility, Vukoja et al. (2018) recently showed that SV and lysosomal proteins are jointly transported in an ARL8-dependent manner within a “presynaptic lysosome-related vesicle” (PLV) in both *Drosophila* larvae and mouse hippocampal neurons. These PLVs correspond to a subpopulation of lysosomes devoid of lysosomal enzymes and are therefore distinct from conventional lysosomes (Vukoja et al., 2018). However, in *C. elegans* DA9 neurons, SV proteins are transported independently of lysosomal proteins, the latter of which do not even enter the axon in these cells (Niwa et al., 2017).

The uncertain relationship of axonal lysosomes and SVPs prompted us to analyze this problem in more detail for mammalian neurons. In particular, we asked, (1) Do lysosomal and SV proteins travel together in the axon of rat and mouse hippocampal neurons? and (2) Is the axonal transport of both types of proteins dependent on BORC? The first question was addressed by tracking the transport of fluorescently tagged lysosomal and SV proteins after photobleaching (PB) of axonal segments, a procedure that allowed us to visualize populations of faint, rapidly moving vesicles that would have otherwise been missed in live-cell imaging experiments. To address the second question, we knocked out the gene encoding the myrlysin subunit of BORC in the mouse and examined the localization and transport of lysosomal and SV proteins in various types of neurons. The results of our studies were surprising in light of previously published work: we found that most lysosomal and SV proteins were transported separately in two types of anterograde vesicles in both rat and mouse hippocampal neurons; in addition, we observed that knockout (KO) of the myrlysin subunit of BORC prevented the transport of lysosomes but not SVPs into the axon of mouse neurons. From these results, we concluded that lysosomes and SVPs are distinct organelles that rely on different machineries for anterograde movement in the axon of mammalian neurons.

## RESULTS

### Lysosomes and SVPs Move Separately in the Axon of Rat Hippocampal Neurons

**Figure 1.**
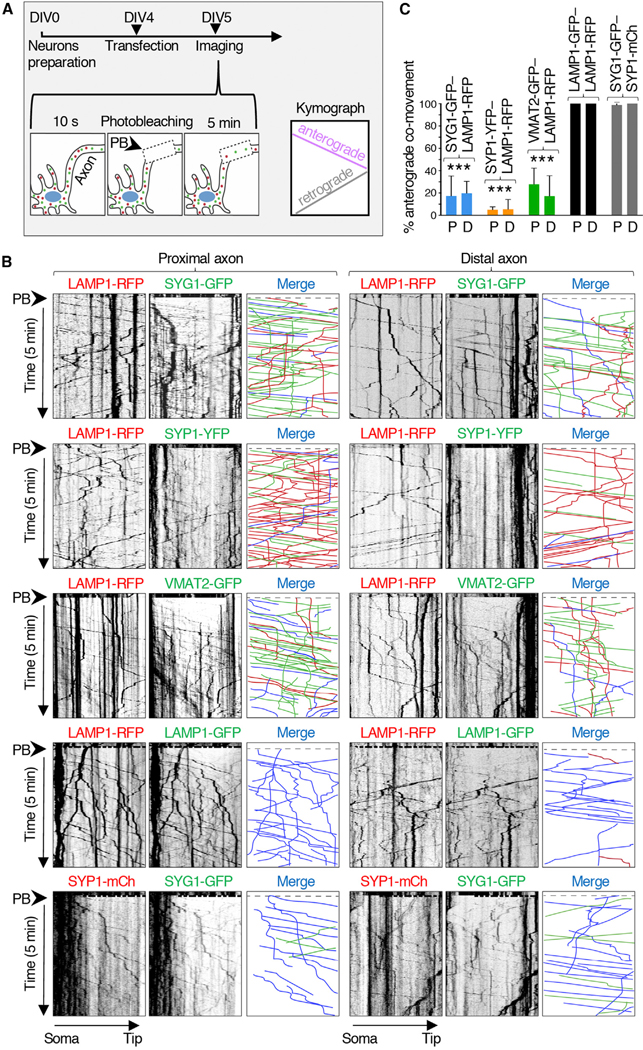
Distinct Sets of Vesicles Mediate the Axonal Transport of Lysosomal and SV Proteins in Rat Hippocampal Neurons (A) Schematic representation of the experimental protocol. DIV4 rat hippocampal neurons were transfected with plasmids encoding lysosomal and/or SV proteins fused to fluorescent proteins and imaged live on a spinning-disk confocal microscope at DIV5. The axon was identified by staining of the AIS with CF640R-conjugated antibody to neurofascin. Fifty micrometer segments of the proximal and distal axon were sequentially recorded every 0.4 s. After 10 s of recording, the axon segments were photobleached (PB) (dashed box), and the recording was continued for an additional 5 min. Kymographs were generated from the videos; lines with negative or positive slopes in the kymographs correspond to vesicles moving in anterograde or retrograde direction, respectively. (B) Kymograph analysis of vesicle movement in the proximal and distal axon of rat hippocampal neurons co-expressing combinations of LAMP1-RFP, SYG1-GFP, SYP1-YFP, VMAT2-GFP, LAMP1-GFP, and SYP1-mCherry (mCh), as indicated in the figure. Single-color images are represented in inverted grayscale. Vertical lines correspond to static foci, which were often brighter than moving vesicles. In the merge panel, red (RFP or mCh) and green (GFP or YFP) lines represent moving vesicles having one or the other marker, and blue lines represent vesicles having both markers. Static vesicles were omitted from the analysis. (C) Quantification of the percentage of anterograde-moving SYG1-GFP, SYP1-GFP, VMAT2-GFP, or LAMP1-GFP vesicles that were also positive for LAMP1-RFP or SYP1-mCh, as indicated. P, proximal axon; D, distal axon. Values are mean ± SD of >100 vesicles in six neurons per condition and are expressed as the percentage of the indicated marker that co-moves with LAMP1-RFP or SYP1-mCh. ***p < 0.005 relative to LAMP1-GFP-LAMP1-RFP co-movement per Student’s t test. See also Figure S1.

To analyze the movement of lysosomes and SVPs in mammalian neurons, we transfected day *in vitro* (DIV) 4 rat hippocampal neurons with combinations of plasmids encoding the lysosomal transmembrane protein LAMP1 (Chen et al., 1985) fused to red fluorescent protein (RFP) and the SV transmembrane proteins synaptogyrin 1 (SYG1) (Stenius et al., 1995), synaptophysin 1 (SYP1) (Wiedenmann and Franke, 1985), or vesicular monoamine transporter 2 (VMAT2) (Liu et al., 1992) fused to green fluorescent protein (GFP) or yellow fluorescent protein (YFP) (Figures 1A and 1B). LAMP1 is concentrated mainly in lysosomes, although significant amounts are also found in early and late endosomes (Fermie et al., 2018), a reflection of its transport to lysosomes via the endocytic pathway (Lippincott-Schwartz and Fambrough, 1987; Janvier and Bonifacino, 2005; Chen et al., 2017). In axons, LAMP1-positive vesicles are heterogeneous, with some having an acidic luminal pH and acid hydrolases (i.e., conventional lysosomes), while others are neutral and lack acid hydrolases (Lee et al., 2011; Gowrishankar et al., 2015; Farías et al., 2017; Cheng et al., 2018; Vukoja et al., 2018; Farfel-Becker et al., 2019). This heterogeneity notwithstanding, for simplicity, herein we refer to all LAMP1-positive vesicles as lysosomes. At 24 h after transfection (i.e., DIV5), the axonal transport of fluorescently tagged lysosomal and SV proteins was imaged live using a spinning-disk confocal microscope (Figure 1A). The axon was identified by staining of the axon initial segment (AIS) with a CF640R-conjugated antibody to neurofascin (Farías et al., 2015). Proximal and distal segments of the axon were imaged for 10 s, after which they were photobleached and further imaged for 5 min (Figures 1A and 1B). PB reduced the intensity of bright, relatively static fluorescent structures that likely corresponded to “orphan synapses” (Krueger et al., 2003) or other organelle clusters along the axon. This methodology enabled the visualization of populations of fainter, rapidly moving vesicles, which were the main focus of our analyses (Video S1). Vesicle movement was analyzed using kymographs such as those shown in Figure 1B.

These analyses showed that vesicles containing LAMP1-RFP (red) or SV proteins fused to GFP or YFP (green) moved in both anterograde (negative slope) and retrograde (positive slope) directions along the axon (Figure 1B). Static foci represented by vertical lines were omitted from our analyses. Anterograde movement was highly processive and occurred with average speeds of 1.5 ± 0.3 μm/s (proximal axon) and 1.7 ± 0.6 μm/s (distal axon) for vesicles containing LAMP1-RFP and 1.8 ± 0.6 to 2.3 ± 0.6 μm/s (proximal axon) and 1.9 ± 0.6 to 2.2 ± 0.8 μm/s (distal axon) for vesicles containing SYG1-GFP, SYP1-YFP, or VMAT2-GFP (Table S1). Importantly for the purpose of our study, only a small fraction of the anterograde vesicles contained both classes of markers (blue lines on the merge kymographs in Figure 1B), while the majority contained either one or the other marker (Figure 1B, red and green lines). Quantification of these data showed that ~5%–25% of vesicles containing SV proteins also contained LAMP1 (Figure 1C). A similarly low degree of co-movement (~22%) was observed for Halo-tagged SYG1 with the lysosomal membrane protein CD63 (Metzelaar et al., 1991) tagged with GFP (Figure S1). As controls, we showed a high degree of co-movement of LAMP1-RFP with LAMP1-GFP, and SYP1-mCherry with SYG1-GFP (Figures 1B and 1C), verifying the specificity of these analyses. These experiments thus demonstrated that in the axon of rat hippocampal neurons, the majority of lysosomal and SV proteins undergo anterograde transport in separate sets of vesicles.

### Redistribution of Lysosomes to the Soma Does Not Affect the Localization of SV Proteins to the Axon of Rat Hippocampal Neurons

**Figure 2.**
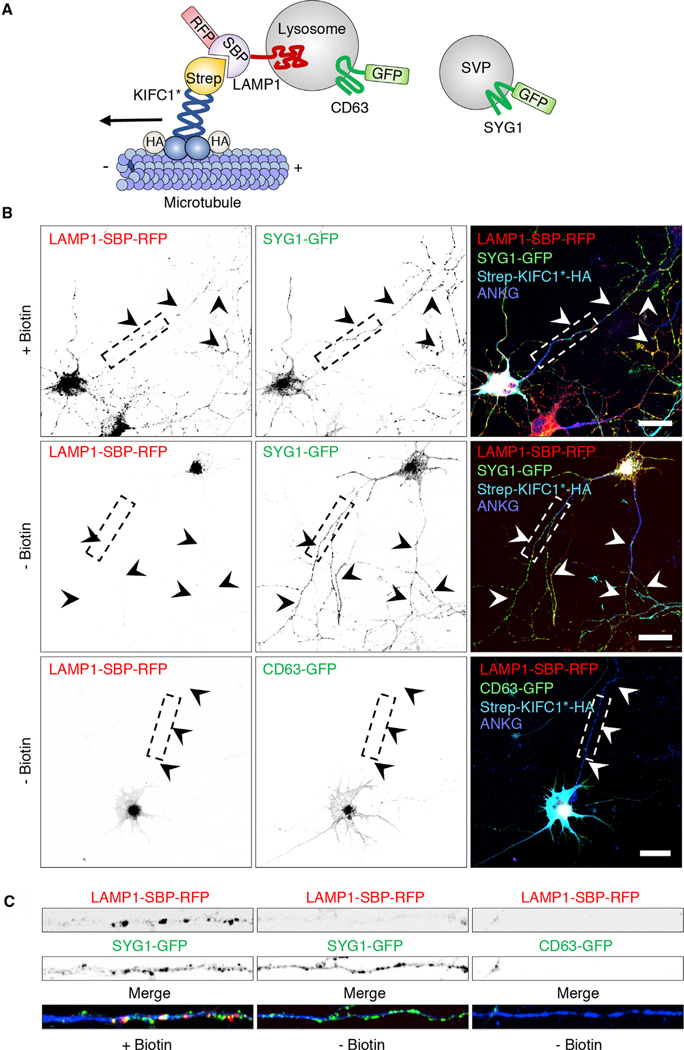
Reversible Association with Motor Proteins (RAMP) Distinguishes Axonal Lysosomes from SVPs (A) Schematic representation of the coupling of lysosomes to the minus-end-directed kinesin KIFC1 using RAMP (Guardia et al., 2019). (B) DIV5 rat hippocampal neurons were transfected with plasmids encoding LAMP1-SBP-RFP and Strep-KIFC1*-HA, together with SYG1-GFP (top and middle rows) or CD63-GFP (bottom row). Neurons were cultured in the presence of NeutrAvidin to remove biotin from the medium and thus enable the SBP-streptavidin interaction to take place. The following day, neurons were incubated for 1 h with (+) or without (−) biotin (in the absence of NeutrAvidin), as indicated in the figure. Neurons were then fixed and immunostained for HA and endogenous ankyrin G (ANKG) (to stain the AIS) and examined using confocal fluorescence microscopy. Scale bars: 20 μm. Arrowheads indicate the axon. (C) Straightened and enlarged 50 mm segments of axons from (B) boxes showing the depletion of LAMP1-SBP-RFP and CD63-GFP, but not SYG1-GFP, from the axon of neurons expressing Strep-KIFC1*-HA in the absence of biotin. In (B) and (C), single-color images are represented in inverted grayscale. See also Figure S2.

To further assess the relationship between lysosomes and SVPs in the axon of rat hippocampal neurons, we used a recently developed method called “reversible association with motor proteins” (RAMP) (Guardia et al., 2019) to cluster lysosomes in the neuronal soma and examine the effect of this manipulation on the presence of SV proteins in the axon (Figure 2A). This approach consisted of co-expressing (1) LAMP1 fused to the streptavidin-binding peptide (SBP) and RFP (LAMP1-SBP-RFP), with (2) streptavidin fused to the motor and coiled-coil domains of the kinesin KIFC1 and the HA epitope (Strep-KIFC1*-HA), together with (3) SYG1-GFP or CD63-GFP (Figure 2A). The choice of the KIFC1 construct was predicated on the ability of this kinesin to move cargo from the plus end to the minus end of microtubules, unlike most other kinesins that move cargo in the opposite direction (Hirokawa et al., 2009; Guardia et al., 2019). The SBP-streptavidin interaction forces coupling of lysosomes to the KIFC1 motor domain, resulting in movement of axonal lysosomes toward the minus end of microtubules in the soma (Guardia et al., 2019). Addition of biotin reverses this interaction, restoring the normal distribution of lysosomes (Guardia et al., 2019). We observed that whereas in the presence of biotin both LAMP1-SBP-RFP and SYG1-GFP localized to the axon (Figures 2B and 2C), in the absence of biotin, only SYG1-GFP remained the axon (Figures 2B and 2C). The axonal localization of another SV protein, VMAT2-GFP, was similarly unaffected by RAMP-mediated redistribution of lysosomes to the soma in the absence of biotin (Figure S2). As a control, we showed that co-expression of LAMP1-SBP-RFP with Strep-KIFC1*-HA depleted another GFP-tagged lysosomal protein, CD63-GFP, from the axon in the absence of biotin (Figure 2B). These experiments further demonstrated that axonal localization of SV proteins is independent from that of lysosomal proteins.

### ARL8 Co-moves with Lysosomes but Not SVPs in the Axon of Rat Hippocampal Neurons

BORC has been shown to promote ARL8-dependent transport of lysosomes in mammalian cells (Pu et al., 2015; Guardia et al., 2016; Farías et al., 2017) and SVPs in *C. elegans* (Zheng et al., 2014; Niwa et al., 2017). To examine whether ARL8 co-moves with lysosomes and/or SVPs in mammalian neurons, we co-transfected DIV4 rat hippocampal neurons with plasmids encoding ARL8B-mCherry (red) and either LAMP1-GFP or SYG1-GFP (green), and at DIV5 we analyzed the movement of vesicles containing these proteins in the distal axon, as described above for Figure 1. We observed that whereas ~90% of anterograde-moving LAMP1-GFP vesicles contained ARL8B-mCherry, only ~10% of anterograde-moving SYG1-GFP vesicles contained ARL8B-mCherry (Figures S3A and S3B). Thus, in the axon of rat hippocampal neurons, anterograde-moving lysosomes were associated with ARL8 to a much greater extent than SVPs.

### Generation of a Myrlysin-KO Mouse to Examine the Requirement of BORC for Axonal Transport of Lysosomes and SVPs

**Figure 3.**
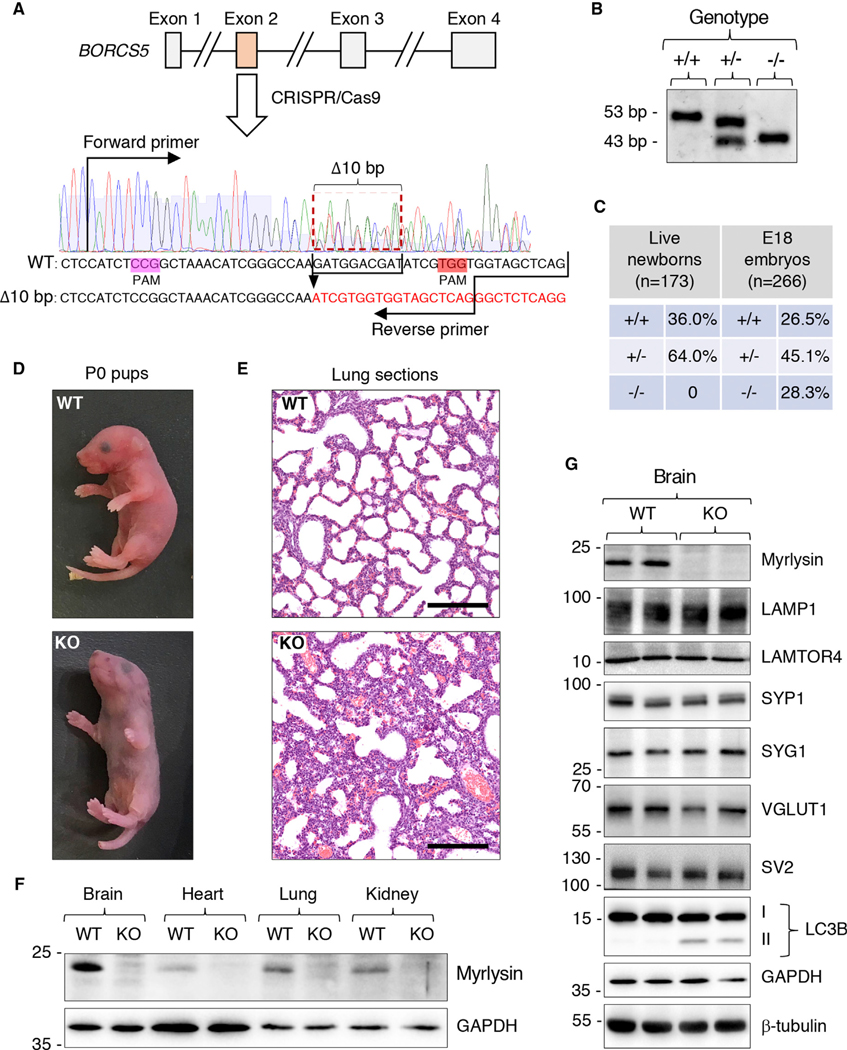
Generation and Characterization of Myrlysin-KO Mouse (A) Schematic representation of the strategy used to disrupt the *BORCS5* gene, encoding myrlysin, by CRISPR-Cas9. Two protospacer-adjacent motif (PAM) sequences were used to delete 10 bp from exon 2. Forward and reverse primers used for genotyping are indicated. The deletion was predicted to cause a frameshift with addition of three extraneous amino acids after amino acid 31 of myrlysin (see also Figure S4). (B) Genotyping of WT (+/+), heterozygous (+/−), and homozygous KO (−/−) alleles generates 53 bp and 43 bp fragments, respectively. (C) Percentages of pups that were alive several hours after birth (live newborns) or embryos collected at E18. n indicates the number of mice analyzed. (D) Pictures of WT and KO pups collected immediately after birth. (E) H&E staining of lung sections from newborn WT and KO pups. Scale bars: 200 mm. (F) SDS-PAGE and immunoblot analysis of different organs from E18 WT and KO mice using antibodies to myrlysin and GAPDH (loading control). (G) SDS-PAGE and immunoblot analysis of brain from two WT and two KO E18 embryos using antibodies to the proteins indicated on the right. In (F) and (G), the positions of molecular mass markers (kDa) are indicated on the left.

To investigate the role of BORC in lysosome and SVP transport in a mammalian organism, we used CRISPR-Cas9 technology to knock out the *BORCS5* gene encoding the myrlysin subunit of BORC in C57BL/6J mouse (Figures 3A and 3B). The mutant mice carried a 10 bp deletion within exon 2 of *BORCS5*, causing a frameshift and the addition of three extraneous amino acids after amino acid 31 (Figures 3A, 3B, and S4). Heterozygous mutant (+/−) mice showed no overt abnormalities, were fertile, and had a normal lifespan. In contrast, no live homozygous KO (−/−) mice (henceforth referred to simply as “KO”) were found in the litters (Figure 3C), and only a few dead pups or body parts were identified as KO a few hours after birth. Collection of the pups immediately after birth allowed us to identify some live KO pups, which exhibited a normal appearance but gasped for air, were cyanotic, and died of respiratory failure within 1 h of birth (Figure 3D). Post-mortem pathology of the KO pups showed atelectasis of the lungs (Figure 3E), confirming that death was caused by an inability to breathe. Genotyping of embryonic day (E) 18 embryos revealed that wild-type (WT) (+/+), heterozygous, and homozygous KO mice were present at expected Mendelian ratios (Figure 3C), consistent with the death of the KO pups occurring soon after birth. We also bred the *BORCS5* mutation for eight generations into the 129X1/SvJ and BALB/cJ mouse strains, with results similar to those in original C57BL/6J strain.

Immunoblot analysis of organs from E18 embryos showed that in WT mice, myrlysin was ubiquitously expressed but was most abundant in the brain (Figure 3F). As expected, myrlysin was absent in all organs from the KO mice (Figure 3F). Further immunoblot analysis showed similar levels of the lysosomal proteins LAMP1 and LAMTOR4 (Sancak et al., 2010) and the SV proteins SYP1, SYG1, VGLUT1 (vesicular glutamate transporter 1) (Bellocchio et al., 2000), and SV2 (SV glycoprotein 2) (Feany et al., 1992) in brains from WT and KO mice (Figure 3G). One difference was the presence of elevated levels of the autophagy protein LC3B-II in KO versus WT mice (Figure 3G). This finding was consistent with decreased degradation of LC3-II due to impaired autophagosome-lysosome fusion, as previously demonstrated in non-neuronal BORC-deficient cells (Jia et al., 2017).

**Figure 4.**
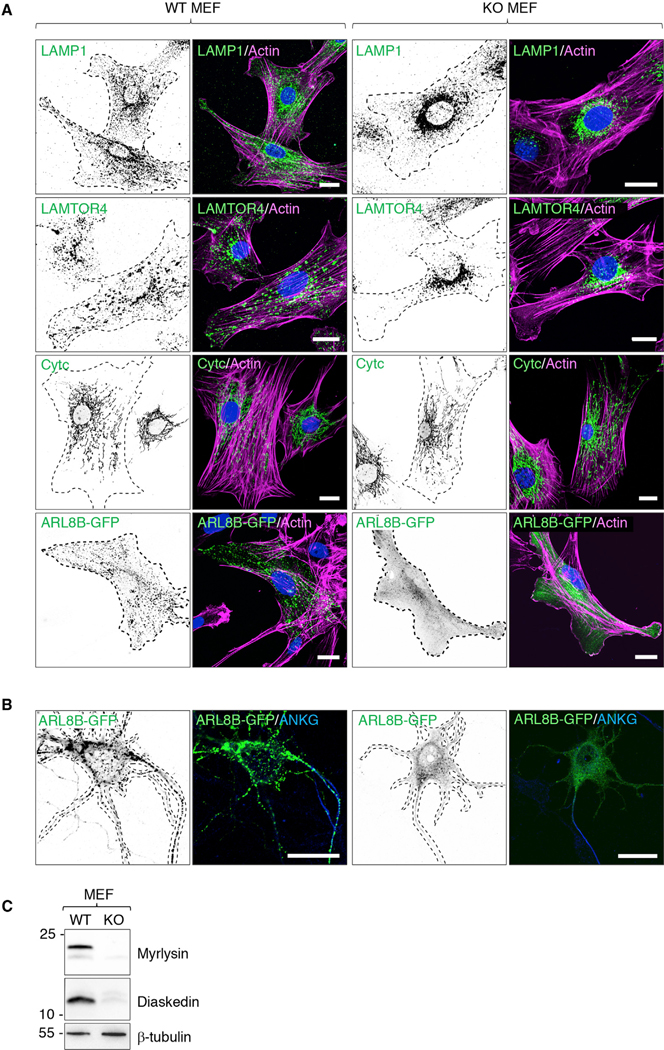
Perinuclear Clustering of Lysosomes and Dissociation of ARL8 in Cells from Myrlysin-KO Mice (A) MEFs from WT and KO E15 embryos were immunostained for endogenous LAMP1 and LAMTOR4 (lysosomes) or cytochrome *c* (Cytc) (mitochondria). Alternatively, MEFs were transiently transfected with a plasmid encoding ARL8B-GFP and immunostained for GFP. Cell edges were outlined by staining of actin with fluorescent phalloidin and indicated by the dashed lines. Nuclei were stained with DAPI. Scale bars: 20 μm. (B) Hippocampal neurons from WT and KO E18 embryos were transfected with a plasmid encoding ARL8B-GFP at DIV 4 and immunostained for ANKG and GFP 1 day later. Cell edges are outlined by dashed lines. Scale bars: 20 μm. (C) SDS-PAGE and immunoblot analysis of WT and KO MEFs using antibodies to myrlysin, diaskedin, and β-tubulin (loading control). The positions of molecular mass markers (kDa) are indicated on the left. In (A) and (B), single-color images are represented in inverted grayscale.

Immunofluorescence microscopy analysis of mouse embryonic fibroblasts (MEFs) from WT and KO mice using antibodies to endogenous LAMP1 and LAMTOR4 (Figure 4A) showed that myrlysin KO caused clustering of lysosomes in the perinuclear area of the cells, similar to that observed in other cell types (Pu et al., 2015; Guardia et al., 2016; Jia et al., 2017; Filipek et al., 2017; Pu et al., 2017). In contrast, the distribution of mitochondria stained for cytochrome *c* (Cytc) was unaltered in the KO MEFs (Figure 4A), confirming the specificity of the lysosomal phenotype. To examine the effect of myrlysin KO on the association of ARL8 with lysosomes, we transfected MEFs (Figure 4A) and E18 hippocampal neurons (Figure 4B) from WT and KO mice with a plasmid encoding ARL8B-GFP. We observed that in both cell types, ARL8B-GFP was associated with lysosomes scattered throughout the cytoplasm in the WT but was largely cytosolic with only a small amount associated with perinuclear lysosomes in the KO (Figures 4A and 4B). These results were consistent with the previously demonstrated role of BORC in recruiting ARL8 to lysosomes. Immunoblot analysis of the MEFs used in these studies confirmed the absence of myrlysin and also showed the loss of another BORC subunit, diaskedin, which was likely due to degradation of the unassembled subunit (Figure 4C). These experiments demonstrated that KO of myrlysin in the mouse recapitulated cellular phenotypes previously observed in cultured cell lines (Pu et al., 2015; Guardia et al., 2016; Jia et al., 2017; Filipek et al., 2017; Pu et al., 2017). The ability to culture neurons from KO embryos enabled subsequent studies of the requirement of BORC for transport of lysosomes and SVPs into the axon.

### Myrlysin KO Prevents Axonal Transport of Lysosomes but Not SVPs in Mouse Hippocampal Neurons

**Figure 5.**
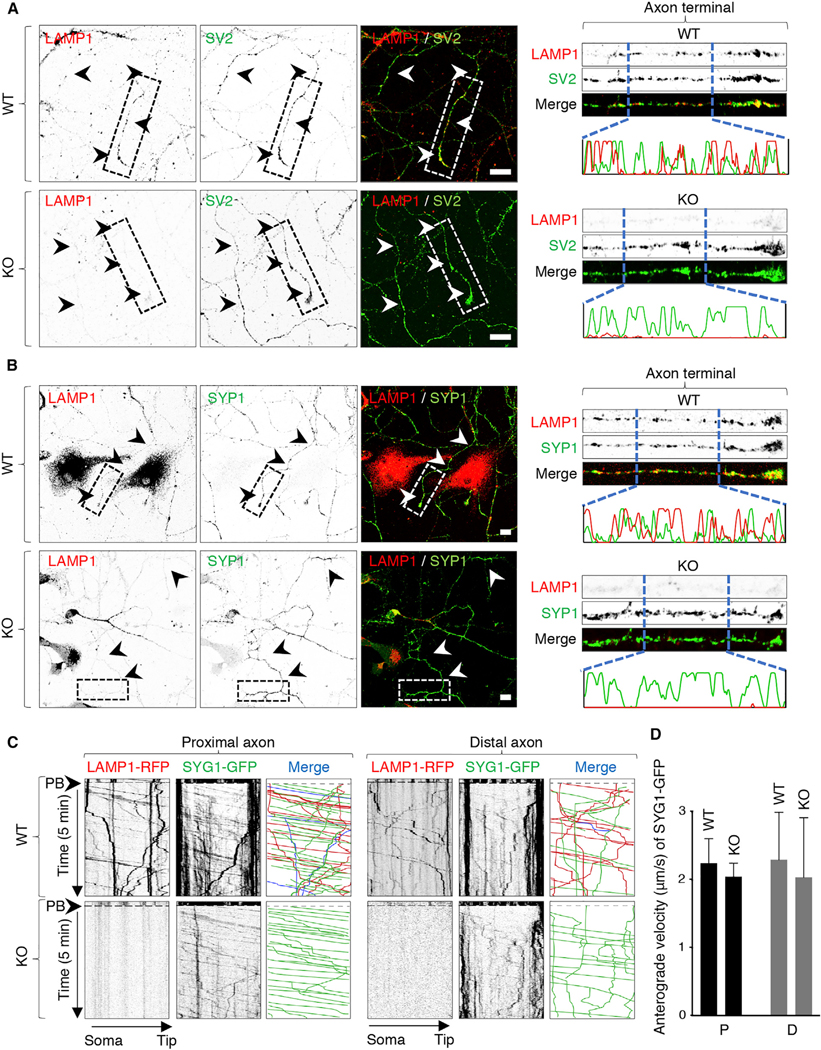
Distinct Effect of Myrlysin KO on the Localization and Movement of Lysosomes and SVPs in the Axon (A and B) DIV5 cultures of hippocampal neurons from WT and myrlysin-KO E18 embryos were immunostained for endogenous LAMP1 together with SV2 (A) or SYP1 (B). Images on the left show single-channel images in inverted grayscale and merged images in color. Arrowheads indicate the axon. Scale bars: 10 μm. Images on the right show straightened 50 μm segments of the distal axon taken from images on the left. Line intensity scans from 15 μm segments are also shown for LAMP1 and SV proteins. (C) Straightened 50 μm segments of the proximal and distal axon from WT and myrlysin-KO E18 hippocampal neurons co-expressing LAMP1-RFP and SYG1-GFP that were analyzed at DIV5 by live-cell imaging and kymographs as described in the legend to Figure 1. Single-channel images are shown in inverted grayscale. In the merge panel, red (RFP) and green (GFP) lines represent moving vesicles having a single marker, and blue lines represent vesicles having both markers. (D) Quantification of the anterograde velocity of SYG1-GFP-positive particles in the proximal (P) or distal (D) axon of WT and KO neurons. Values are mean ± SD of >100 vesicles in six neurons per condition and are not statistically different. See also Figure S5.

Immunofluorescent staining and confocal microscopy of DIV5 hippocampal neurons from E18 WT mice showed the presence of both endogenous LAMP1 and SV2 in the axon (Figure 5A, left panels). Line scanning of the axon showed overlapping but distinct distributions of these markers (Figure 5A, right panels). Similar observations were made for endogenous LAMTOR4 and SV2 in DIV6 rat hippocampal neurons analyzed using confocal microscopy (Figure S5A) and transgenic CD63-GFP and SYP1-mCherry in DIV14 rat hippocampal neurons analyzed using structured illumination microscopy (SIM) (Figure S5B). Importantly, in DIV5 hippocampal neurons from KO mice, LAMP1 was depleted from the axon, but SV2 was not (Figure 5A). Similarly, myrlysin KO did not alter the axonal localization of endogenous SYP1 (Figure 5B). Additional immunofluorescence microscopy analyses showed co-localization of pre-synaptic SYP1 with post-synaptic PSD-95 (Sampedro et al., 1981) along the dendrites of DIV12 hippocampal neurons from both WT and KO mice (Figure S5C), indicating that SYP1 was able to reach axon terminals in more mature neurons irrespective of the presence or absence of myrlysin.

Although informative of the distribution of lysosomal and SV proteins, the above analyses could not distinguish moving vesicles from static structures. To further analyze the requirement of BORC for axonal transport of lysosomes and SVPs, we transfected hippocampal neurons from WT and KO mice with plasmids encoding LAMP1-RFP and SYG1-GFP. PB and live-cell imaging of proximal and distal segments of the axon from WT mice showed that, as in rat hippocampal neurons (Figure 1B), LAMP1-RFP and SYG1-GFP traveled mostly in separate vesicles (Figure 5C), with only ~17%–20% showing co-movement of both markers. Moreover, we observed that myrlysin KO prevented transport of LAMP1-RFP but not SYG1-GFP into the axon (Figure 5C). The loss of myrlysin also had no detectable effect on the anterograde velocity of SYG1-GFP-positive vesicles in both the proximal and distal axon (Figure 5D). These experiments further demonstrated that axonal lysosomes and SVPs are distinct organelles and that they also exhibit distinct dependence on BORC for anterograde movement in mouse hippocampal neurons.

### Depletion of Lysosomal but Not SV Proteins in Axon-Enriched Areas of the Brain of Myrlysin-KO Mice

**Figure 6.**
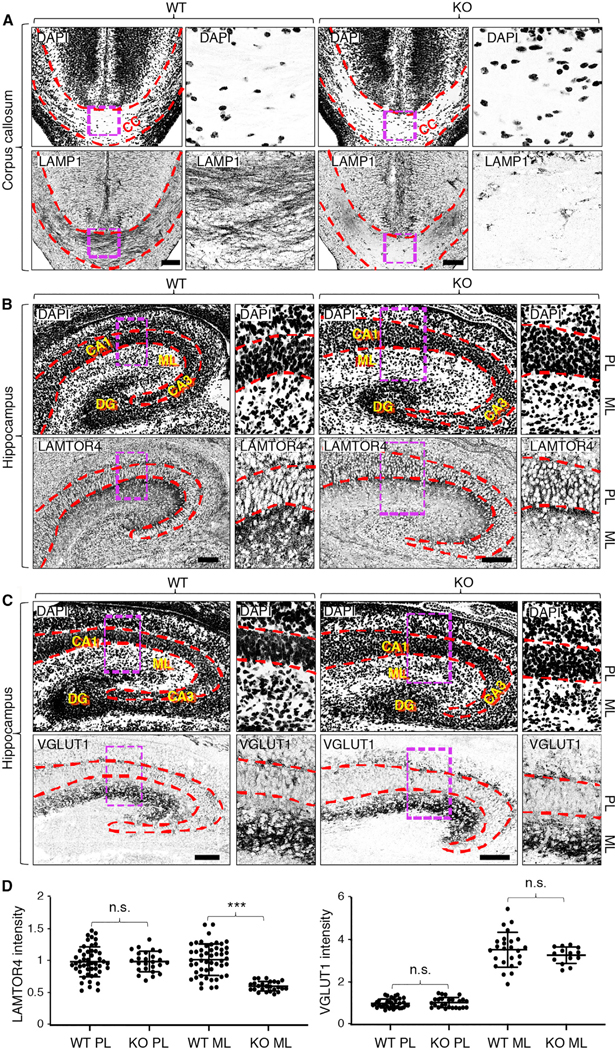
Decreased Staining for Lysosomal but Not SV Proteins in the Corpus Callosum and Molecular Layer of the Hippocampus in Myrlysin-KO Brains (A) Coronal sections of brains from WT and myrlysin-KO mouse E18 embryos were stained for endogenous LAMP1. Nuclei were stained with DAPI. Boxes are magnified on the corresponding right panels. The dashed red lines demarcate the boundaries of the corpus callosum (CC). (B and C) Coronal sections of brains from WT and myrlysin-KO mouse E18 embryos mice stained for endogenous LAMTOR4 (B) or VGLUT1 (C). Nuclei were stained with DAPI. The dashed red lines demarcate the pyramidal layer (PL) of the hippocampus, including the cornu ammonis 1 and 3 (CA1 and CA3) regions. The molecular layer (ML) and dentate gyrus (DG) are also indicated. Images are shown in inverted grayscale. Scale bars: 100 μm. (D) Quantitative analysis of LAMTOR4 and VGLUT1 intensity in the PL and ML of hippocampi from (B) and (C). The mean ± SD of fluorescence in different areas from two brains per condition is indicated. Statistical significance per Student’s t test: ***p < 0.005; n.s., not significant.

Next, we investigated if myrlysin KO altered the distribution of endogenous lysosomal and SV proteins in brain sections from E18 mice. In regions of the brain enriched in neuronal cell bodies, we found it difficult to discern if myrlysin KO had any effect on lysosome distribution. Therefore, we focused on the corpus callosum, which is composed mainly of myelinated axons connecting the two cerebral hemispheres and is relatively devoid of neuronal cell bodies (Figure 6A, top row, DAPI staining). We observed that whereas in WT brain the corpus callosum exhibited many parallel fibers (i.e., axons) that were strongly positive for LAMP1, in KO brain the corpus callosum showed little LAMP1 staining that was restricted mostly to a few glial cells (Figure 6A, bottom row). We also examined the hippocampus, where neuronal somata are most concentrated in the pyramidal layer (PL) of the cornu ammonis (CA1 and CA3 regions) and in the dentate gyrus (DG) (Figures 6B and 6C, top rows, DAPI staining). Axons and dendrites, on the other hand, are enriched in the molecular layer (ML), located in the interior of the hippocampus (Figures 6B and 6C, top rows). Staining for the lysosomal marker LAMTOR4 showed no appreciable difference in the PL and DG of WT and KO hippocampi (Figure 6B, bottom row). However, staining for LAMTOR4 was drastically reduced in the ML of KO relative to WT hippocampi (Figure 6B, bottom row). Staining for the SV marker VGLUT1, which is enriched in glutamatergic pre-synaptic terminals in the ML, on the other hand, showed no difference in WT and KO hippocampi (Figure 6C, bottom row). Quantification of these observations confirmed the reduced staining for LAMTOR4 but not VGLUT1 in the ML of KO hippocampi (Figure 6D). These findings are consistent with the depletion of lysosomes but not SVPs from the axons of myrlysin-KO neurons *in situ*, as previously observed in cultured neurons (Figure 5).

### Depletion of Lysosomal but Not SV Proteins in the Neuromuscular Junction of Myrlysin-KO Mice

**Figure 7.**
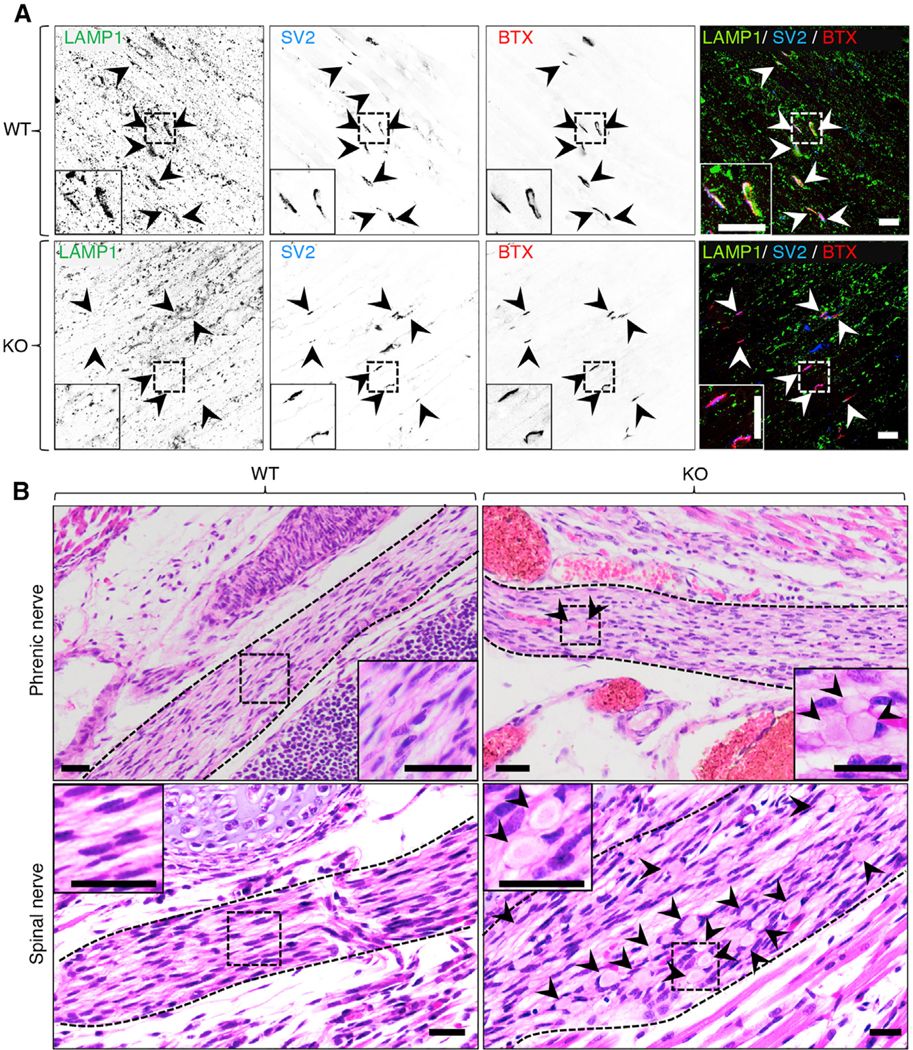
Decreased Staining for Lysosomal but Not SV Proteins at Diaphragm Neuromuscular Junctions and Evidence of Neuroaxonal Dystrophy in Myrlysin-KO Embryos (A) Whole diaphragms were harvested from WT and myrlysin-KO E18 embryos and co-stained with antibodies to LAMP1 and SV2 and with Alexa Fluor 594-conjugated α-bungarotoxin (BTX). Single-channel images are shown in inverted grayscale. Arrowheads point to individual NMJs. Scale bars: 20 μm. Notice the absence of LAMP1 staining in KO NMJs. See also Figure S6. (B) H&E staining of sagittal sections from whole WT and KO E18 embryos. The dashed lines demarcate the boundaries of the phrenic and spinal nerves. Arrowheads indicate dystrophic eosinophilic bodies corresponding to swollen axons. Scale bars: 200 μm. Insets in (A) and (B) are magnified views of the boxed areas.

The death from respiratory failure of the KO mice suggested a possible defect in the function of the diaphragm, which is innervated by the phrenic nerve at specialized synapses known as neuromuscular junctions (NMJs). To analyze the status of NMJs, whole mounted diaphragms from WT and KO E18 mouse embryos were immunostained with antibodies to endogenous LAMP1 and SV2, and with Alexa Fluor 594-conjugated α-bungarotoxin (BTX), a fluorescent toxin probe that binds post-synaptic acetylcholine receptors (AChRs) at the NMJ. In WT diaphragms, we observed coincident LAMP1, SV2, and BTX staining at NMJs (Figure 7A, top row, arrowheads). In KO diaphragms, on the other hand, LAMP1 was absent, but SV2 and BTX were still present at the NMJ (Figure 7A, bottom row, arrow-heads). The presence of SYP1 at diaphragm NMJs was similarly unaffected in the KO mice (Figure S6). Neither the number nor the appearance of NMJs showed any other difference in WT and KO mouse diaphragms. From these results, we concluded that the absence of BORC prevents transport of lysosomes, but not SVPs or AChRs, to the NMJ. Failure of the newborn KO mice to breathe is thus not likely due to defective SVP or AChR transport but to other neurological or pulmonary problems.

### Evidence of Neuroaxonal Dystrophy in the Phrenic and Spinal Nerves of Myrlysin-KO Mice

Although SV proteins were normally localized to the NMJ, we hypothesized that the absence of lysosomes could affect the structure and/or function of axons in the myrlysin-KO mice. Indeed, hematoxylin and eosin (H&E) staining of tissue sections from E18 embryos revealed the presence of dystrophic eosinophilic bodies corresponding to swollen axons in the phrenic and spinal nerves from KO but not WT mice (Figure 7B). These findings are consistent with the absence of lysosomes resulting in neuroaxonal dystrophy, which in the phrenic nerve could be the cause of respiratory failure in the KO mice.

## DISCUSSION

In this study we attempted to meld observations made in different organisms, such as *C. elegans*, *Drosophila*, and rodents, into a comprehensive model for the axonal transport of lysosomal and SV proteins in mammalian neurons. On the basis of previous studies already described in the Introduction, we tested two alternative hypotheses: (1) that axonal lysosomes and SVPs are one and the same organelle and (2) that axonal lysosomes and SVPs are distinct organelles, but both depend on BORC for coupling to kinesin motors. As it turned out, neither hypothesis was supported by our data. We found that in rat and mouse hippocampal neurons, axonal lysosomes and SVPs are distinct organelles that move independently of each other; however, BORC is required for axonal transport of lysosomes but not SVPs.

### Independent Transport of Lysosomal and SV Proteins in the Axon of Rat and Mouse Neurons

Our live-cell imaging of hippocampal neurons from rat and mouse embryos showed that lysosomal and SV transmembrane proteins tagged with fluorescent proteins undergo anterograde transport largely in different sets of vesicles. This conclusion applies to the lysosomal proteins LAMP1 and CD63, and at least three SV proteins, SYG1, SYP1, and VMAT2. In addition, we found that ARL8B co-moved with LAMP1 but not SYG1. Moreover, depletion of lysosomes from the axon using RAMP had no effect on the axonal localization of SYG1 and VMAT2. Finally, KO of the myrlysin subunit of BORC prevented the axonal localization and transport of the lysosomal proteins LAMP1 and LAM-TOR4, but not the SV proteins SYG1, SYP1, VGLUT1, and SV2 in various neuronal types. Our findings are therefore at variance with those of a previous study showing that >85% of anterograde vesicles carrying the SV protein VGLUT1 also contained LAMP1 in mouse hippocampal neurons at DIV4, a time in culture similar to that in most of our experiments (DIV5) (Vukoja et al., 2018). In our experiments, the extent of lysosomal-SV protein co-movement was at most ~25%. Although we do not know the reason for these discrepancies, we speculate that they may arise from the different methodologies were used to image axonal transport. In particular, we found it important to perform PB before imaging, to allow visualization of populations of faint, small, fast-moving vesicles that may be the carriers of lysosomal and SV proteins in the axon. Without PB, imaging was confounded by the presence of brighter, larger puncta that may correspond to clusters of organelles along the axon. These puncta often contained both lysosomal and SV proteins but were largely static, and when they moved, they did so for relatively short distances and at relatively low speeds. Our findings are consistent with recent observations that axonal lysosomes and SVPs have different dynamics in cultured rat hippocampal neurons (Guedes-Dias et al., 2019). Our findings also rule out that most SVPs “hitchhike” on lysosomes, as recently shown for RNA granules (Cioni et al., 2019; Liao et al., 2019). From these experiments we concluded that in mammalian neurons, axonal lysosomes and SVPs are largely distinct, independently moving organelles.

### BORC Is Required for Lysosome but Not SVP Transport in the Axon of Mouse Hippocampal Neurons

Having shown that lysosomes and SVPs are distinct organelles, we nevertheless expected that they would both depend on BORC for coupling to kinesins in the axon of mammalian neurons, as is the case for lysosomes in non-neuronal mammalian cells (Pu et al., 2015; Guardia et al., 2016; Jia et al., 2017; Filipek et al., 2017; Pu et al., 2017) and SVPs in *C. elegans* neurons (Zheng et al., 2014; Niwa et al., 2017). However, analysis of neurons from WT and myrlysin-KO mice showed that BORC was required for lysosome but not SVP transport into the axon. This conclusion was drawn from analyses of hippocampal neurons in primary culture, as well as neurons in the corpus callosum, the ML of the hippocampus, and the diaphragm NMJ. The inhibitory effect of myrlysin KO on lysosome transport was in line with our previous findings that short hairpin RNA (shRNA)-mediated knockdown (KD) of myrlysin or lyspersin in rat hippocampal neurons reduced transport of lysosomes into the axon (Farías et al., 2017). The lack of an effect of myrlysin KO on SVP transport in mouse neurons highlighted important differences in the mechanism of SVP coupling to kinesin in *C. elegans*, which is indeed dependent on BORC (Zheng et al., 2014; Niwa et al., 2017). This difference extends to ARL8, which functions downstream of BORC for lysosome transport in mammals (Pu et al., 2015; Guardia et al., 2016) and SVP transport in *C. elegans* (Zheng et al., 2014; Niwa et al., 2017) and *Drosophila* (Vukoja et al., 2018). In our experiments, ARL8B did not co-localize with SVPs in WT rat neurons, and its shift to the cytosol in myrlysin-KO mouse cells did not impede SVP transport into the axon. Although we cannot rule out subtle effects, our findings indicate that BORC is largely dispensable for SVP transport into the axon of mouse neurons.

### Alternative Mechanisms for Axonal SVP Transport in Mammalian Neurons

If BORC is not involved, then how are SVPs coupled to kinesins in mammals? The phosphoinositide-binding pleckstrinhomology (PH) domain at the C terminus of *C. elegans* kinesin-3 UNC-104 was previously shown to be necessary for axonal SV transport, probably through direct binding to acidic phospholipids on the SV membrane (Klopfenstein and Vale, 2004; Choudhary et al., 2017). However, this interaction is unlikely to suffice for attachment of SVPs to UNC-104, as BORC and ARL8 are required for axonal transport of SVPs (Klassen et al., 2010; Wu et al., 2013; Zheng et al., 2014; Niwa et al., 2016, 2017). This suggests the existence of other adaptors for coupling SVPs to kinesins in mammalian neurons. Previous work showed that a protein named DENN/MADD functions both as a GEF and an effector of the small GTPase RAB3 to couple RAB3-carrying vesicles to the kinesin-3 proteins KIF1A and KIF1Bβ for axonal transport in mammalian neurons (Niwa et al., 2008). Furthermore, organelles immunoisolated with anti-KIF1A beads contained both RAB3A and SV markers (Okada et al., 1995). However, KO of the *C. elegans* DENN/MAD ortholog AEX-3 did not alter axonal transport of the SV-localized synaptotagmin 1 ortholog SNT-1 (Iwasaki et al., 1997), and RAB3-KO mice exhibited normal SV biogenesis and transport (Schlüter et al., 2004), suggesting that RAB3 and other SV proteins are transported in different carrier vesicles. Several other adaptors were also shown to participate in coupling of vesicles carrying SV proteins (mainly SNAREs) to kinesin-1 motors in both *C. elegans* and mammalian neurons. These include *C. elegans* UNC-14 (Sakamoto et al., 2005) and its distant mammalian relative NESCA/RUSC1 (MacDonald et al., 2012), the *C. elegans* UNC-16 ortholog of the mouse JIP3/JSAP and *Drosophila* Sunday Driver (SYD) protein (Sakamoto et al., 2005; Byrd et al., 2001), the mammalian FEZ1 and *C. elegans* UNC-76 orthologs (Chua et al., 2012), the mammalian protein syntabulin (Su et al., 2004), and a molecular network involving the small GTPase RAB21, its GEF VARP, and the spectraplakin MACF1 (Burgo et al., 2012). However, at least in *C. elegans*, mutations in kinesin-1 alter SV protein localization by mechanisms other than impairment of long-range movement in the axon (Byrd et al., 2001; Yan et al., 2013). Moreover, it is currently unclear if these adaptors couple different types of SVPs to kinesins. Ascertaining the function of all of these adaptors in axonal transport of SVPs in mammalian neurons will require testing of their requirement in the same model system. One such system could be the recently developed human i3neurons, which are readily amenable to gene KO and KD, and to perform analyses of axonal transport (Wang et al., 2017; Tian et al., 2019; Boecker et al., 2019).

### Potential Causes of Lethality in BORC-KO Mice

Newborn myrlysin-KO pups had apparently normal anatomical features but died from respiratory failure soon after birth. Mice with null mutations in the gene encoding the diaskedin (BORCS7) subunit of BORC were also recently reported to die in the neonatal period, although in this case the cause of death was not determined (Snouwaert et al., 2018). Interestingly, mice with a spontaneously arising truncating mutation in diaskedin were viable but developed progressive axonal dystrophy and motor function impairment reminiscent of human hereditary spastic paraplegia (HSP) (Snouwaert et al., 2018), consistent with a requirement for BORC in neuronal function. Also relevant to this discussion are the observations that mice with KO of genes encoding KIF1Bβ (Zhao et al., 2001) and KIF5A (Xia et al., 2003) die from respiratory failure after birth. All of these observations suggest that a defect in axonal transport might be responsible for the neonatal lethality of myrlysin-KO mice. It remains to be determined, however, the deficiency of which organelle causes this lethal phenotype. We observed that myrlysin-KO mice have apparently normal transport and localization of SV proteins, particularly at the phrenic nerve-diaphragm NMJ, which controls respiration. We cannot rule out, however, that the synapses are somehow abnormal, either because of a subtle defect in SV protein localization or because of a role of lysosomes in the maintenance of synaptic function. In this latter regard, lysosome exocytosis was previously shown to promote axon outgrowth in rat corticostriatal neurons (Martinez-Arca et al., 2001). Moreover, a recent study showed that the synaptic organizer Cbln1 is exocytosed from axonal lysosomes into the axon terminal for activity-dependent synapse modification in cerebellar granular cells (Ibata et al., 2019). Finally, lysosomal function is required for the clearance of damaged proteins or organelles in the axon, thus contributing to the maintenance of synaptic function (Boecker and Holzbaur, 2019). Failure of these lysosomal functions might explain the neuroaxonal dystrophy observed in the phrenic and spinal nerves, as well as the neonatal death, of the myrlysin-KO mice.

A recent study revealed the presence of a single-nucleotide, splice-acceptor variant (NM_058169.4:c.203–1G>T) of *BORCS5* in a patient with global developmental delay, corpus callosum agenesis, seizures, polymicrogyria, and abnormality of the cerebral cortex (Charng et al., 2016). On the basis of our studies on the myrlysin-KO mouse, we think that impaired transport of lysosomes into the axon could contribute to the neuropathogenesis of this disorder.

An alternative cause for the respiratory failure of newborn myrlysin-KO mice could be a primary lung defect. In this regard, zebrafish with a mutation in the BLOS1 (or BLOC1S1) protein, a shared subunit of BORC (Pu et al., 2015) and the related complex BLOC-1 (Starcevic and Dell’Angelica, 2004), were unable to inflate their swim bladder, the teleost equivalent of the mammalian lung (Chen et al., 2018). This phenotype was caused by a defect in the surfactant system that allows the swim bladder to expand. Importantly, lung lamellar bodies that synthesize and secrete pulmonary surfactant in mammals are lysosome-related organelles that share some lysosomal components and biogenetic pathways with lysosomes (Bowman et al., 2019). It is thus also possible that the neonatal death of myrlysin-KO mice is due to lung abnormalities, including defective surfactant secretion. The distinction between a neurological and a pulmonary defect as the cause of death of myrlysin-defective mice will have to await the development of a tissue-specific myrlysin KO.

### Concluding Remarks

The axonal transport of lysosomal and SV proteins in separate vesicles and by different mechanisms demonstrated here allows specific regulation of the transport of each set of proteins depending on the needs of the neuron. Our findings thus provide a framework for future studies on the control of lysosome and SVP transport and on the maturation and function of these organelles as they move toward the axon terminal in mammalian neurons.

## STAR★METHODS

Detailed methods are provided in the online version of this paper and include the following:

### RESOURCE AVAILABILITY

#### Lead Contact

Further information and requests for resources and reagents should be directed to and will be fulfilled by the Lead Contact, Juan S. Bonifacino (juan.bonifacino@nih.gov).

#### Materials Availability

Plasmids not covered by any restrictions like MTAs generated in this study will be available upon request. Myrlysin-KO mouse line generated in this study will be available upon request.

#### Data and Code Availability

This study did not generate any unique datasets or code.

### EXPERIMENTAL MODEL AND SUBJECT DETAILS

- All animal procedures were conducted following the NIH Guide for the Care and Use of Laboratory Animals, under protocols #19–011 (rats) and #18–036 (mice) approved by the NICHD Animal Care and Use Committee.
- Mice and rats used in the study were naive, i.e., they were not treated with any drugs.
- All the animals used in this study had normal health status. The animal facility is regularly checked for standard pathogens.
- Multiple independent experiments were carried out using several biological replicates specified in the figure legends. Animals of both genders were used.
- Pregnant albino rats were delivered to our facility on day 17 of gestation. They were housed under a 12-h light-dark cycle for 24 h. The following day, the animals were sacrificed by carbon dioxide inhalation followed by decapitation prior to embryo extraction and preparation of neurons. Hippocampal neurons from embryos of the same litter were pooled and analyzed. Neuronal cultures were randomly allocated to different treatments (staining, transfection with plasmids, etc.).
- Mice were housed in groups or kept individually for timed pregnancies under a 12-h light-dark cycle. To time the deliveries, we housed single male and female mice in separate cages. The female’s weight was recorded, and she was transferred overnight into the cage housing the male for no longer than 12 h. The following day, the two animals were separated. After one week, we monitored the weight of the female for increases greater that 1.5 g. When the pregnancy was successful, we harvested embryos after 15 days for preparation of MEFs, or after 18 days for culture of primary neurons. Hippocampal neurons of mice with identical genotype and from the same litter were pooled and analyzed.

### METHOD DETAILS

#### Culture of Primary Hippocampal Neurons

Primary hippocampal neurons were prepared from rats and mice as previously described (Farías et al., 2016). In brief, embryonic day 18 (E18) rats or mice were harvested and euthanized by decapitation. The brain was removed from the skull, and hippocampi were dissected in fresh Hank’s medium and treated with 0.25% trypsin (GIBCO) for 15 min at 37°C. The tissue was then washed twice with Hank’s medium and resuspended in plating medium consisting of Dulbecco’s Modified Eagle Medium (DMEM) without phenol red, supplemented with 4.5 g/L glucose, 25 mM HEPES, 10% heat-inactivated horse serum (GIBCO), 100 U/mL penicillin and 100 μg/mL streptomycin. The hippocampi were then disrupted mechanically with a glass Pasteur pipet whose tip was narrowed to around 50% of its original diameter. The cells were counted, and 80,000 cells plated on 18-mm glass coverslips previously coated with polylysine (Sigma) and 5 μg/mL laminin (Roche). After 4 h, the medium was changed to complete Neurobasal medium (CNB) consisting of Neurobasal medium (GIBCO), supplemented with 1X B27 serum free (Thermo Scientific), 4.5 g/L glucose, and 100 U/mL penicillin-streptomycin (GIBCO).

#### Transfection and Immunofluorescence Microscopy of Cultured Cells

Primary rat and mouse hippocampal neurons (DIV4) or MEFs (24 h after plating) grown on 18-mm coverslips were transfected with 0.8–3 μg plasmid DNA per coverslip, using 1.3 μL Lipofectamine 2000 reagent (Invitrogen) diluted in 200 mL Opti-MEM I (GIBCO) and 800 μL Neurobasal medium for neurons or Opti-MEM I for MEFs. Approximately 1 h after transfection, the medium was replaced by fresh medium. Cells were then cultured for 24 h before fixation or live-cell imaging. Alternatively, rat hippocampal neurons were transfected at DIV13 using a calcium phosphate procedure, in which 0.8–3 μg plasmid DNA, 2.5 M CaCl_2_ and HEBS buffer (137 mM NaCl, 4.7 mM KCl, 7.5 mM glucose, 21 mM HEPES, 0.7 mM Na_2_HPO_4_) were mixed and added to neurons. After 1 h, neurons were washed twice with washing solution (10 mM HEPES, pH 7.3, and HEBS), and the medium was replaced. Cells were fixed for 18 min with 4% paraformaldehyde (PFA), 4% sucrose, in phosphate-buffered saline (PBS) supplemented with 0.1 mM CaCl_2_ and 1 mM MgCl_2_ (PBS-CM). Cells were then washed twice in PBS-CM and permeabilized with 0.2% v/v Triton X-100 for 15 min at room temperature. Cells were incubated with PBS-CM containing 0.2% gelatin (blocking solution) for 20 min. Primary and secondary antibodies were diluted in blocking solution and sequentially incubated for 30 min at 37°C. Coverslips were mounted with Fluoromount-G (Electron Microscopy Sciences).

#### Live-cell Imaging of Hippocampal Neurons by Spinning-Disk Confocal Microscopy

Movement of vesicles positive for lysosome and SVP markers was visualized in neurons transfected at DIV4 as described above. Neurons were co-transfected with plasmids encoding LAMP1-RFP along with plasmids encoding SYG1-GFP, SYP1-YFP, VMAT2-GFP or LAMP1-GFP. Alternatively, plasmids encoding CD63-GFP, SYG1-Halo, SYP-mCherry or ARL8B-mCherry were used. Axonal co-movement was imaged live at DIV5. To identify the axon initial segment (AIS), rat neurons were stained with a CF640R (Biotium)-conjugated antibody to the AIS protein neurofascin. Each channel was sequentially exposed for 200 ms with no interval delay. After 10 s of baseline recording, photobleaching (PB) was performed for both channels to facilitate identification of moving vesicles, and neurons were recorded for an additional 5 min. Kymographs were generated with Fiji. Lines of one-pixel thickness and 50-mm length were tracked in the axonal PB region and straightened, followed by stack re-slicing and Z-projection. The number of individual and co-movement events was determined manually from kymographs. Particles moving in anterograde and retrograde directions appear as lines with negative and positive slopes, respectively. Live-cell imaging was performed on a spinning-disk Eclipse Ti Microscope System (Nikon) equipped with a humidified environmental chamber kept at 37°C and 5% CO_2_. Confocal images were taken with a Plan Apo VC 60x objective (N.A. 1.40) and a high-speed electron-multiplying charge-coupled device camera (iXon Life 897; Andor). NIS-Elements AR microscope imaging software was used for acquisition.

#### Reversible Association with Motor Proteins (RAMP)

DIV5 rat hippocampal neurons were transfected with plasmids encoding LAMP1-SBP-RFP and Strep-KIFC1*-HA, together with SYG1-GFP, CD63-GFP or VMAT2-GFP (Figure 2A). Neurons were cultured in the presence of 0.5 mg/mL NeutrAvidin, to remove biotin from the medium, and thus enable the SBP-streptavidin interaction. The following day, neurons were incubated for 1 h in the presence of 50 μM D-biotin, or left untreated (in the absence of NeutrAvidin). Neurons were then fixed and immunostained for HA and endogenous ankyrin-G, and examined by confocal fluorescence microscopy.

#### Confocal Imaging and Structured Illumination Microscopy

Confocal microscopy images were collected using a Zeiss LSM 780 confocal microscope with a Plan Apochromat 63x objective (N.A. 1.40), using settings recommended by the manufacturer. Structured illumination microscopy (SIM) of fixed cells was performed on a Zeiss Elyra PS.1 microscope with a Plan-Apochromat 63× 1.4 N.A. objective lens at room temperature. Five orientation angles of the excitation grid with five phases each were acquired for each z-plane. Images were then reconstructed with the SIM module in Zeiss ZEN Black software using automatic settings.

#### Myrlysin-KO Mouse Generation

Myrlysin-KO mice were generated by The Jackson Laboratory (https://www.jax.org/). Two single guide RNAs (sgRNAs), targeting exon 2 of the *BORCS5* gene encoding myrlysin (http://www.informatics.jax.org/marker/MGI:1915024), were designed to introduce frameshifting indels using CRISPR-Cas9. The two sgRNAs used have the following sequence: sgRNA1: 5ʹ-ATCTTGGCCCGATGTT TAGC-3ʹ, sgRNA2: 5ʹ-GGCTAAACATCGGGCCAAGA-3ʹ, and utilize CGG and TGG as protospacer adjacent motifs (PAM), respectively (Figure 3A). WT Cas9 was then injected, together with the sgRNAs, into C57BL/6J zygotes. Embryos were transferred into WT C57BL/6J pseudopregnant female mice and, 3 weeks after birth, tail biopsies from pups were tested by sequencing to identify founder mice. This technique generates mosaic animals, in which different cells have different mutations (https://www.jax.org/jax-mice-and-services/model-generation-services/custom-model-services/mosaicism). Naturally, zygotes experiencing different rates of mutation success will exhibit varying degrees of mosaicism, and so the resulting animals may display different degrees of physiological impairment. The presence of different mutations was determined by TIDE software (https://tide.nki.nl/). From the first round of injection, three founders were identified, two of which were runt. The third animal did not appear runt and carried the following indel frequency of mutations: 38 bp deletion (~20%), 10 bp deletion (36.7%), 1 bp insertion (6.2%) and 2 bp insertion (23.8%). This founder was bred with WT C57BL/6J and the progeny (F1) had the following mutations in heterozygosity: 38 bp deletion, 10 bp deletion and 1 bp insertion. To be passed onto the F1 generation, the mutation must occur in germline cells, explaining why the 2 bp insertion was not present in the progeny. Four of the F1 generated animals carrying the 10 bp deletion were shipped to our laboratory. After breeding the F1 founders with WT animals and expanding our colony, we mated heterozygous animals. The generated litters always presented a few dead animals and body parts, together with healthy littermates. After genotyping, we confirmed that the dead animals always corresponded to the homozygous KO. The percentage of dead homozygous KO mice was less than 25%, suggesting that other homozygous KO mice were cannibalized by the mothers. Observation of several deliveries confirmed that the KO always died neonatally. When we bred heterozygous mice and genotyped the embryos, we observed the expected Mendelian ratios of the different genotypes (Figure 3C), indicating that the KO animals were generated with normal rates but failed to survive the immediate neonatal period. To exclude a role of the genetic background in the neonatal lethality of homozygous myrlysin-KO mice, we backcrossed C57BL/6J +/− mice into 129X1/SvJ or BALB/cJ WT mice for 8 generations. Mating of 8^th^ generation +/− 129X1/SvJ or BALB/cJ mice resulted in a similar phenotype of neonatal death of homozygous −/− pups, indicating that this phenotype is independent of the mouse strain used.

#### Genotyping of Myrlysin-KO and WT Mice

The genotype of WT (+/+), homozygous KO (−/−) and heterozygous (+/−) mice was determined by polymerase chain reaction (PCR) using primers for sequences within exon 2 (Figure 3A). Deletion of 10 bp (GATGGACGAT) from exon 2, is predicted to cause a frame-shift after amino acid 31 and the addition of 3 extraneous amino acids at the C terminus of the truncated protein (Figure S4). The different alleles were identified by isolating genomic DNA from ear or tail snips. Primers with the following sequences were designed to amplify the regions flanking the deletion in exon 2: 5ʹ-CATCTCCGGCTAAACATCGGGC-3ʹ and 5ʹ-CTGAGCTACCACCACG ATATC-3ʹ. Using these primers, we amplified a 53 bp fragment from the WT allele and a 43 bp fragment from the deleted allele (Figure 3B). DNA amplification was performed using an initial denaturation (95°C for 3 min) followed by 25 cycles of denaturation (95°C, 15 s), annealing (60°C, 15 s) and extension (72°C, 5 s). To extract the genomic DNA and for PCR reactions, we used the KAPA Mouse Genotyping Kit (KAPA Biosystems). PCR products were separated using 5% agarose gels.

#### Preparation of Tissue Extracts and Immunoblotting

Organs were harvested from WT or myrlysin-KO E18 mice and snap-frozen in dry ice. They were then homogenized in 150 mM NaCl, 10 mM HEPES pH 7.5, 1 mM EDTA, 1x protease inhibitor cocktail (Roche) and 1% v/v Triton X-100, using a 1 mL glass mortar. Homogenized organs were centrifuged at 16,000 x g, for 10 min, and the supernatant was transferred for Bradford (BioRad) protein assay. Alternatively, MEFs were scraped from the plate in PBS pH 7.5, 0.5% v/v Triton X-100 supplemented with protease inhibitors, incubated on ice for 30 min, and centrifuged at 16,000 x g for 10 min. Twenty mg from each protein extract was heated in Laemmli sample buffer (Bio-Rad) containing 2.5% v/v 2-mercaptoethanol (Sigma-Aldrich) at 95°C for 5 min, and then resolved by SDS-PAGE. Gels were subsequently blotted onto nitrocellulose membrane and blocked with 5% BSA in Tris-buffered saline, 0.1% v/v Tween 20 (TBS-T) for 20 min. Membranes were sequentially incubated with primary antibody and secondary HRP-conjugated antibody diluted in TBS-T. Supersignal West Dura Reagents (Thermo Scientific) was used for detection of the antibody signal with a BioRad Chemidoc MP imaging system. Controls for sample loading were either GAPDH or β-tubulin.

#### Culture of Primary Mouse Embryonic Fibroblasts

To prepare primary mouse embryonic fibroblasts (MEFs), WT and E15 KO mice were sacrificed, minced and trypsinized for 15 min in 5 mL of 0.25% trypsin (Corning) at 37°C. The tissue was homogenized twice by passing it through a serological 10-mL pipet. Trypsinization was interrupted by adding 8 mL of DMEM, supplemented with 2 mM L-glutamine (GIBCO), 10% fetal bovine serum (GIBCO), 100 U/mL penicillin-streptomycin (GIBCO) (complete DMEM). Cells were spun down at 900 x g for 5 min, and the pellet was resuspended in 10 mL complete DMEM and seeded onto 10 cm cell culture dishes. After 6 h, the medium was replaced. When confluent, the cells were split at 1:3 ratio, and then frozen or seeded onto 12-well plates for immunofluorescence and immunoblot analyses.

#### Immunohistochemistry of WT and KO Mouse Embryonic Tissues

Brain tissues were harvested from E18 WT or myrlysin-KO embryos and fixed overnight at 4°C using 4% PFA in PBS. Tissues were then immersed in 30% w/v sucrose-PBS at 4°C for a few days, until organs sank. The tissues were next frozen in Optimal Cutting Temperature (OCT) compound and cut into 25 mm sections using a Thermo Fisher HM-525 NX cryostat, maintaining the sample and the working space at −20°C. Once collected directly onto Superfrost glass slides (coated with a solution of 5% w/v gelatin, 0.5% w/v chromium potassium sulfate dodecahydrate (Sigma), and dried for 24 h), sections were incubated with PBS at room temperature (RT) for 5 min. Antibodies to VGLUT1 were used to identify SVs, and antibodies to LAMP1 or LAMTOR4 were used to identify lysosomes. Primary antibodies were diluted in fresh carrier solution (0.5% v/v Triton X-100, 1% v/v goat serum in PBS). Sections were incubated in blocking solution (20% v/v goat serum, 80% v/v carrier solution) for 1 h at room temperature prior to staining overnight with primary antibody at 4°C. Sections were subsequently washed several times in carrier solution, and incubated with their respective secondary antibodies at room temperature for 1 h. Following a PBS wash and 3 additional washes in carrier solution, the sections were stained with 14 μM DAPI in PBS to identify nuclei. Lastly, each slide was washed with PBS (room temperature, for 5 min) and mounted onto a glass coverslip using Fluoromount-G (Southern Biotech). Sections were imaged on a Zeiss LSM 780 microscope at 63x magnification and stitched using ZEN Black software.

#### Whole-Mount Diaphragm Staining for NMJ Analysis

WT and KO mouse embryos were harvested at E18 and sacrificed. The diaphragm was isolated by carefully removing all the organs in the abdominal cavity and cutting the connection with the ribcage. Once isolated, the diaphragm was fixed overnight using 4% PFA in PBS at 4°C. The following day, the organ was rinsed in PBS and pinned-stretched on a Sylgard-coated plate. This provides a better access of the antibodies to deeper areas of the muscle. The diaphragm was than permeabilized and blocked for 1 h in 0.5% v/v Triton X-100 in PBS (PBT) containing 2% w/v BSA and 4% v/v goat serum. Primary antibody to LAMP1, SV2 or SYP1 was diluted in PBT containing 2% w/v BSA and 4% v/v goat serum. The antibody was forced into the muscle by pipetting for 10 min, making sure the pipet tip did not directly touch the tissue. Tissues were then incubated overnight at 4°C with primary antibodies. PBT was used to rinse the tissues 3 times for 1 h each. Secondary antibodies and Alexa Fluor 594-conjugated α-Bungarotoxin (BTX), were incubated following the same method and solutions used for the primary antibody. After overnight incubation at 4°C, diaphragms were rinsed with PBT and mounted flat on a microscope slide under a coverslip in Fluoromount-G (Electron Microscopy Science).

#### Histology of Whole Embryos

For histological analysis, E18 littermates were harvested and genotyped to identify WT and KO animals. The embryos were fixed overnight using 4% w/v PFA in PBS at 4°C. The following day, samples were washed three times in PBS and sent for pathology analysis. Paraffin embedding, sectioning and H&E staining were performed by Histoserv Inc. (Germantown, MD, https://www.histoservinc.com/services/he).

### QUANTIFICATION AND STATISTICAL ANALYSIS

A strategy for randomization, stratification or blind selection of samples was not carried out. No statistical methods were used to predetermine sample sizes. Instead, multiple independent experiments were carried out using several sample replicates as detailed in the figure legends. Quantified data were analyzed using Prism7 software (GraphPad, San Diego, CA). All bar graphs in the figures represent the mean SD from multiple determinations. Each experiment was reproduced at least three times on different days. The statistical significance of differences between two samples was calculated using Student’s t test. Statistical significance for groups was calculated by one-way analysis of variance (ANOVA). Significance is denoted using asterisks: *p < 0.05, **p < 0.005, and ***p < 0.005. p > 0.05 is not significant (ns). The total number of samples (n) analyzed in each experiment is indicated in the figure legends.

## Supplementary Material

1

2

3

## Figures and Tables

**Table:** KEY RESOURCES TABLE

REAGENT or RESOURCE	SOURCE	IDENTIFIER
Antibodies		

Myrlysin (BORCS5/ LOH12CR1), rabbit, IB 1:500	Proteintech	Cat# 17169–1-AP; RRID:AB_2137150
Diaskedin (BORCS7/C10orf32), rabbit, IB 1:500	Abnova	Cat# PAB23142; RRID:AB_11122571
GAPDH (0411), HRP-conjugated, IB 1:300	Santa Cruz	Cat# sc-47724; RRID:AB_627678
LAMP1, rat, IB 1:500, IF 1:500, IHC 1:100	Developmental Studies Hybridoma Bank	Cat# 1D4B; RRID:AB_2134500
LAMTOR4 (C7orf59) (D6A4V), rabbit, IB 1:500, IF 1:500, IHC 1:500	Cell Signaling	Cat# 12284; RRID:AB_2797870
Synaptophysin 1 (SYP1) (D-4), mouse, IB 1:1,000	Santa Cruz	Cat# sc-17750; RRID:AB_628311
Synaptophysin 1 (SYP1) (H-93), rabbit, IF 1:500, IHC 1:500	Santa Cruz	Cat# sc-9116; RRID:AB_2199007
Synaptogyrin 1 (SYG1), rabbit, IB 1:500	Synaptic Systems	Cat# 103002; RRID:AB_887818
Vesicular glutamate transporter 1 (VGLUT1), guinea pig, IB 1:500, IF 1:250, IHC 1:500	Millipore Sigma	Cat# AB5905; RRID:AB_2301751
Synaptic vesicle glycoprotein 2A (SV2), mouse, IB:200, IF: 1:200, IHC 1:500	Developmental Studies Hybridoma Bank	Cat# SV2; RRID:AB_2315387
Autophagy-related protein LC3B, rabbit, IB 1:10,000	Sigma-Aldrich	Cat# L7543; RRID:AB_796155
β-tubulin, rabbit, IB 1:1,000	Cell Signaling	Cat# 2146; RRID:AB_2210545
Cytochrome-C (Cytc) (6H2.B4), mouse, IF 1:1,000	BD Bioscience	Cat# 556432; RRID:AB_396416
HA (influenza hemagglutinin) epitope tag, chicken, IF 1:250	Millipore Sigma	Cat# AB3254; RRID:AB_91371
Ankyrin-G (P-20), goat, IF 1:50	Santa Cruz	Cat# sc-31778; RRID:AB_2289736
Ankyrin-G, mouse, IF 1:20	NeuroMab	Cat# 73–146; RRID:AB_10697718
Pan-Neurofascin (extracellular), mouse, IF 1:1,000	UC Davis/NIH NeuroMab Facility	Cat# 75–172, RRID:AB_2282826
GFP polyclonal antibody, chicken, IF 1:500	Thermo Fisher	Cat# A10262; RRID:AB_2534023
Alexa Fluor 488-conjugated donkey anti-rabbit IgG, IF 1:1,000	Thermo Fisher	Cat# A21206; RRID:AB_2535792
Alexa Fluor 546-conjugated donkey anti-rabbit IgG, IF 1:1,000, IHC 1:1000	Thermo Fisher	Cat# A10040; RRID:AB_2534016
Alexa Fluor 488-conjugated donkey anti-mouse IgG, IF 1:1,000	Thermo Fisher	Cat# A21202; RRID:AB_141607
Alexa Fluor 647-conjugated goat anti-mouse IgG, IHC 1:1,000	Thermo Fisher	Cat# A21235; RRID:AB_2535804
Alexa Fluor 488-conjugated donkey anti-rat IgG, IF 1:1000, IHC 1:1,000	Thermo Fisher	Cat# A21208; RRID:AB_2535794
Alexa Fluor 568-conjugated goat anti-rat IgG, IF 1:1000, IHC 1:1,000	Thermo Fisher	Cat# A11077; RRID:AB_2534121
Alexa Fluor 488-conjugated goat anti-Guinea pig IgG, IHC 1:1,000	Thermo Fisher	Cat# A11073; RRID:AB_2534117
Alexa Fluor 647-conjugated goat anti-chicken IgY, IF 1:1,000	Thermo Fisher	Cat# A21449; RRID:AB_2535866
Alexa Fluor 488-conjugated goat anti-chicken IgY, IF 1:1,000	Thermo Fisher	Cat# A32931; RRID:AB_2762843
Alexa Fluor 647-conjugated donkey anti-goat IgG, IF 1:1,000	Thermo Fisher	Cat# A21447; RRID:AB_2535864
Alexa Fluor 405-conjugated goat anti-chicken IgY (H+L), IF 1:1,000	Abcam	Cat# AB175675; RRID:AB_2810980
HRP-conjugated goat anti-rabbit IgG (H+L), IB 1:5,000	Jackson ImmunoResearch	Cat# 111–035-144; RRID:AB_2307391
HRP-conjugated goat anti-rat IgG (H+L), IB 1:5,000	Jackson ImmunoResearch	Cat# 112–035-143; RRID:AB_2338138
HRP-conjugated donkey anti-mouse IgG (H+L), IB 1:5,000	Jackson ImmunoResearch	Cat# 715–035-150; RRID:AB_2340770
HRP-conjugated donkey anti-Guinea pig IgG (H+L), IB 1:5,000	Jackson ImmunoResearch	Cat# 706–035-148; RRID:AB_2340447

Chemicals, Peptides, and Recombinant Proteins		

Lipofectamine 2000	Thermo Fisher	Cat# 11668019
Alexa Fluor 594-conjugated α-Bungarotoxin	Thermo Fisher	Cat# B35451
CF®640R Mix-n-Stain antibody labeling kit	Biotium	Cat# 92245
Alexa Fluor 647-Phalloidin	Thermo Fisher	Cat# A22287
Janelia Fluor®−549 Halo Tag Ligand	Gift from Luke Lavis, HHMI Janelia, USA	N/A
NeutrAvidin Protein	Thermo Fisher	Cat# 31050
D-biotin	Sigma-Aldrich	Cat# 47868

Experimental Models: Cell Lines		

Primary hippocampal neuron cultures from rat E18 brains	N/A	N/A
Primary hippocampal neuron cultures from WT and myrlysin-KO mouse E18 brains	N/A	N/A
Mouse embryonic fibroblasts (MEFs) from WT and myrlysin-KO E15 mice	N/A	N/A

Experimental Models: Organisms/Strains		

Mice: myrlysin-KO 10 bp deletion	Made by The Jackson Laboratory	N/A
Mice: C57BL/6J	The Jackson Laboratory	Stock No: 000664 | Black 6
Rats: Sprague Dawley® outbred	ENVIGO	Stock No: Sprague Dawley® SD®

Oligonucleotides		

sgRNA #1: 5ʹ-ATCTTGGCCCGATGTTTAGC-3ʹ	This study	N/A
sgRNA #2: 5ʹ-GGCTAAACATCGGGCCAAGA-3ʹ	This study	N/A
Primer: transgenic N0 genotyping forward: 5ʹ-ACTACAAACTCCTCTTTCTAATGACTG-3ʹ	This study	N/A
Primer: transgenic N0 genotyping reverse: 5ʹ-CTTCAGACAGGGCATTCAGG-3ʹ	This study	N/A
Primer: 10 bp deletion genotyping forward: 5ʹ-CATCTCCGGCTAAACATC GGGC —3ʹ	Eurofins Genomics	N/A
Primer: 10 bp deletion genotyping reverse: 5ʹ- CTGAGCTACCACCACGATATC-3ʹ	Eurofins Genomics	N/A

Recombinant DNA		

Modified Clontech plasmid-LAMP1-RFP	Addgene; Sherer et al., 2003; deposited by Walther Mothes, Yale, USA	Cat# 1817
pEGFP-N1-LAMP1-GFP	Farías et al., 2017	N/A
pEGFP-N1-SYG1-GFP	Zhao and Nonet, 2001	N/A
pEGFP-N1-SYG1-Halo	This study	N/A
pEGFP-N1-SYP1-YFP	Gift from Ann Marie Craig, UBC, Canada	N/A
pmCherry-N1-SYP1-mCherry	Gift from Ann Marie Craig, UBC, Canada	N/A
pEGFP-N1-VMAT2-GFP	This study	N/A
pmCherry-N1-ARL8B-mCherry	Farías et al., 2017	N/A
pcDNA3.1/CT-GFP-ARL8B-GFP	Kaniuk et al., 2011	N/A
Modified Clontech Plasmid-LAMP1-SBP-RFP	Guardia et al., 2019	N/A
Modified Clontech pmCherry-N1-Strep-KIFC1*-HA	Guardia et al., 2019; deposited in Addgene	Cat# 120171
pEGFP-N1-CD63-GFP	Farías et al., 2017	N/A

Software and Algorithms		

Fiji/ImageJ	NIH	https://fiji.sc/
TIDE software	Free online software	https://tide.nki.nl/

IF: immunofluorescence, IB: immunoblotting, IHC: immunohistochemistry.

## Highlights

- Lysosomes and synaptic vesicle precursors (SVPs) move separately in mammalian axons
- BORC is required for transport of lysosomes but not SVPs in mammalian axons
- BORC-subunit knockout causes axon swellings and neonatal death in mice
- Defective axonal lysosome transport may underlie pathology of human BORC deficiency
