# Supplementary material for: Synaptic Vesicle Precursors and Lysosomes Are Transported by Different Mechanisms in the Axon of Mammalian Neurons
Source: Cell Rep. Author manuscript; Available in PMC 2020 Sep 8. (PMC7478246; doi:10.1016/j.celrep.2020.107775)
Supplement: 1 [file NIHMS1604811-supplement-1.pdf]

Cell Reports, Volume *31*

Supplemental Information

Synaptic Vesicle Precursors and Lysosomes Are Transported by Different Mechanisms in the Axon of Mammalian Neurons

Raffaella De Pace, Dylan J. Britt, Jeffrey Mercurio, Arianne M. Foster, Lucas Djavaherian, Victoria Hoffmann, Daniel Abebe, and Juan S. Bonifacino

Fig. S1

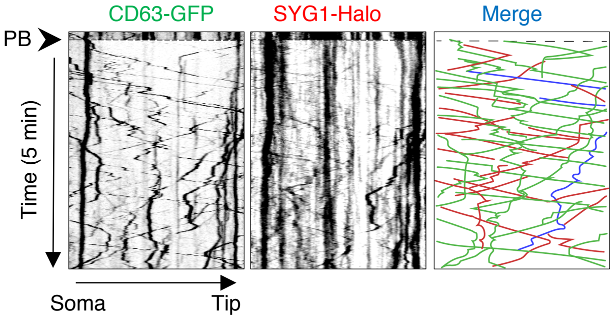

Fig. S2

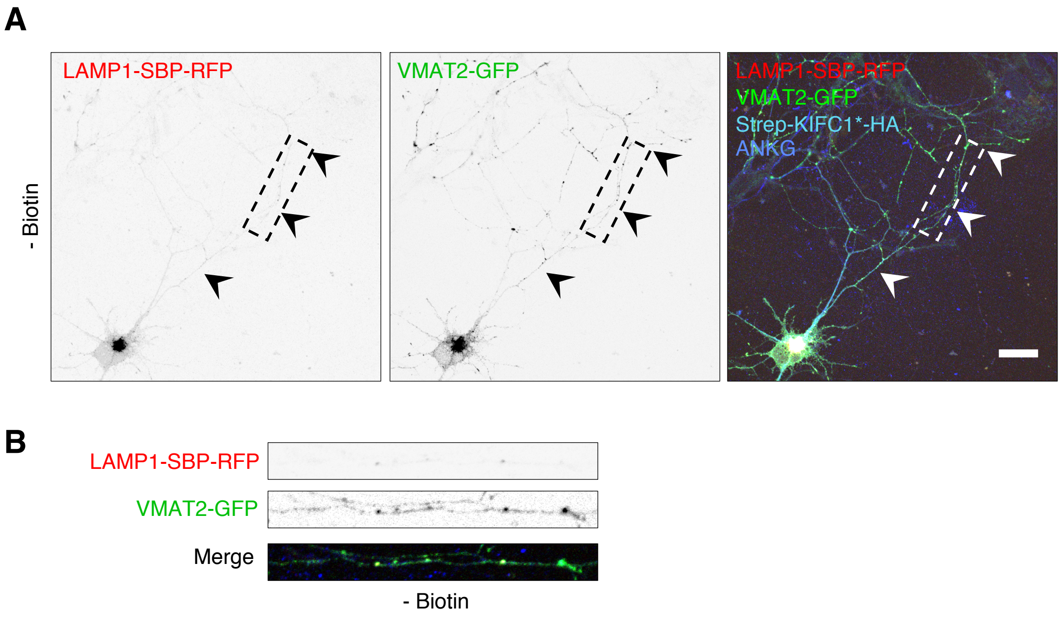
A
- Biotin
LAMP1-SBP-RFP
VMAT2-GFP
LAMP1-SBP-RFP
VMAT2-GFP
Strep-KIFC1*-HA
ANKG
B
LAMP1-SBP-RFP
VMAT2-GFP
Merge
- Biotin

Fig. S3

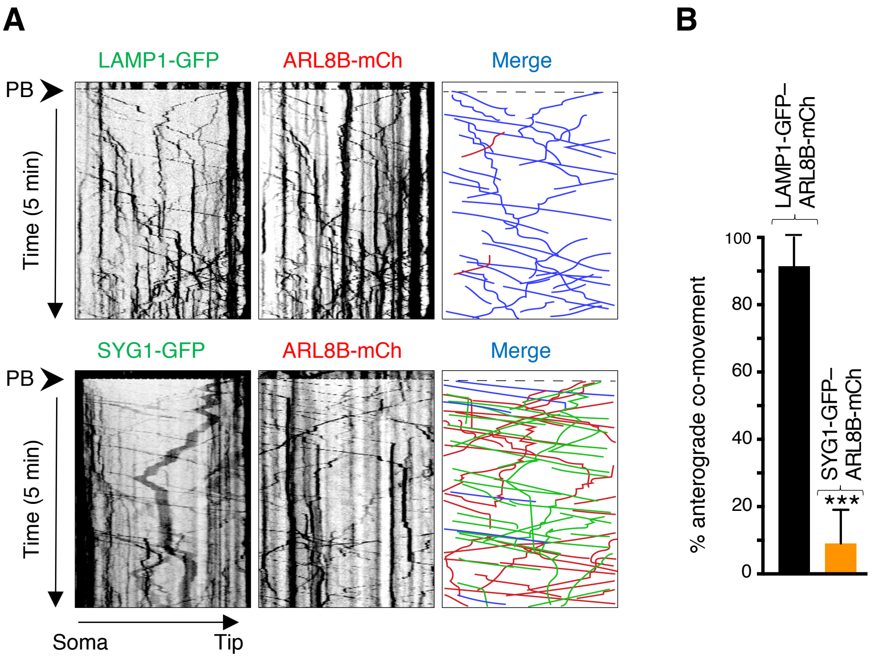
A
LAMP1-GFP
ARL8B-mCh
Merge
PB
Time (5 min)
SYG1-GFP
ARL8B-mCh
Merge
PB
Time (5 min)
Soma
Tip
B
% anterograde co-movement
100
80
60
40
20
0
LAMP1-GFP–
ARL8B-mCh
SYG1-GFP–
ARL8B-mCh
***

Fig. S4

```
                                          20                                        40
                                           |                                         |
WT myrlysin  MPVPLRTATL  VYRHLEPRKQ  VTPSPAKHRA  KMDDIVVVAQ  GSQASRNVSN  50
KO myrlysin  MPVPLRTATL  VYRHLEPRKQ  VTPSPAKHRA  KSWW*-----  ----------  35

                              60                                        80                                       100
                               |                                         |                                         |
WT myrlysin  DPDVIKLQEI  PTFQPLLKGL  LSGQTSPTNA  KLEKLDSQQV  LQLCLRYQDH  100
KO myrlysin  ----------  ----------  ----------  ----------  ----------

                                         120                                       140
                                           |                                         |
WT myrlysin  LHQCAEAVAF  DQNALVKRIK  EMDLSVETLF  CFMQERQKRY  AKYAEQIQKV  150
KO myrlysin  ----------  ----------  ----------  ----------  ----------

                             160                                       180
                               |                                         |
WT myrlysin  NEMSAILRRI  QMGIDQTVPL  MERLNSMLPE  AERLEPFSMK  PERERH*  197
KO myrlysin  ----------  ----------  ----------  ----------  -------
```

Fig. S5

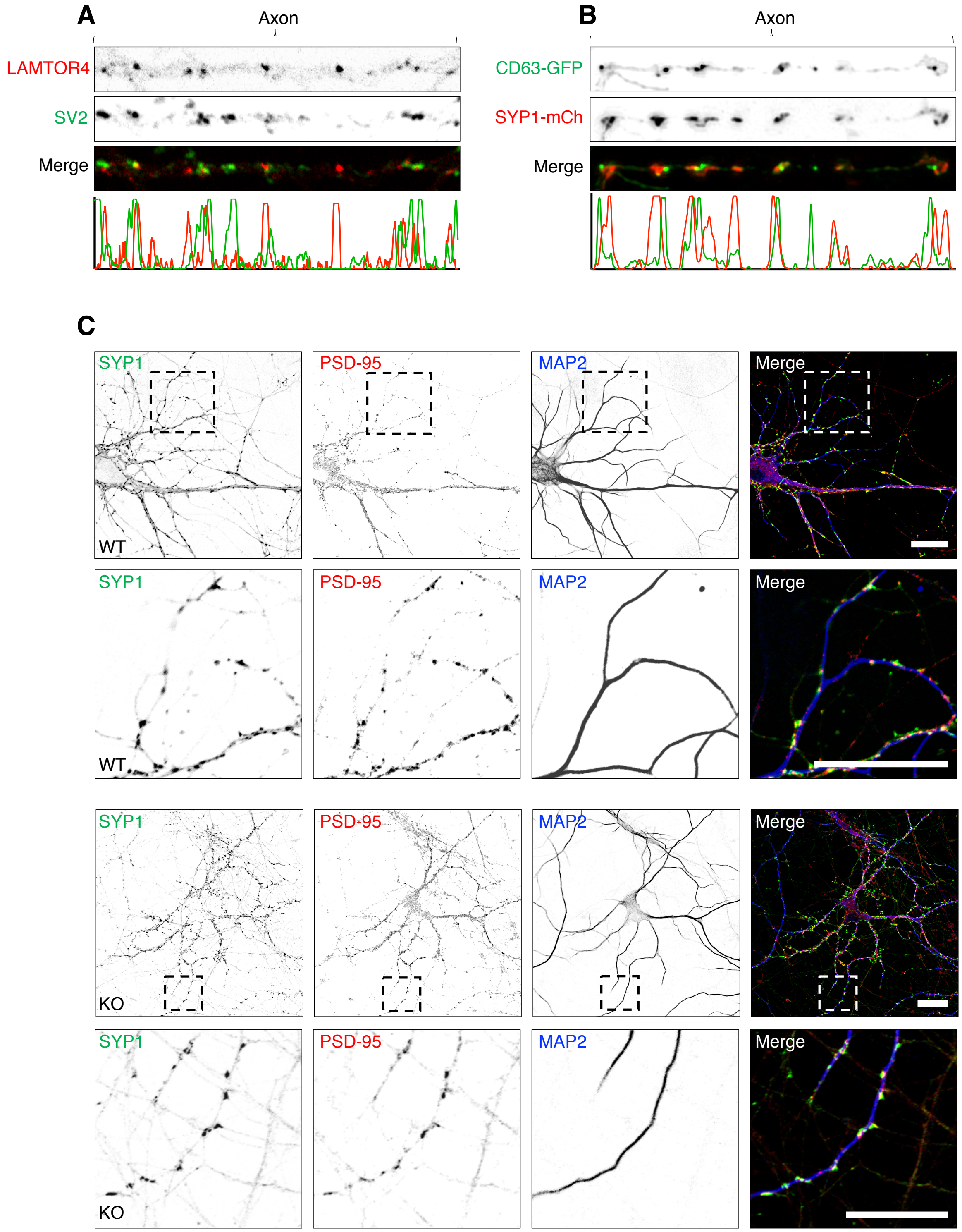

A
Axon
LAMTOR4
SV2
Merge
B
Axon
CD63-GFP
SYP1-mCh
Merge
C
SYP1
PSD-95
MAP2
Merge
WT
KO

Fig. S6

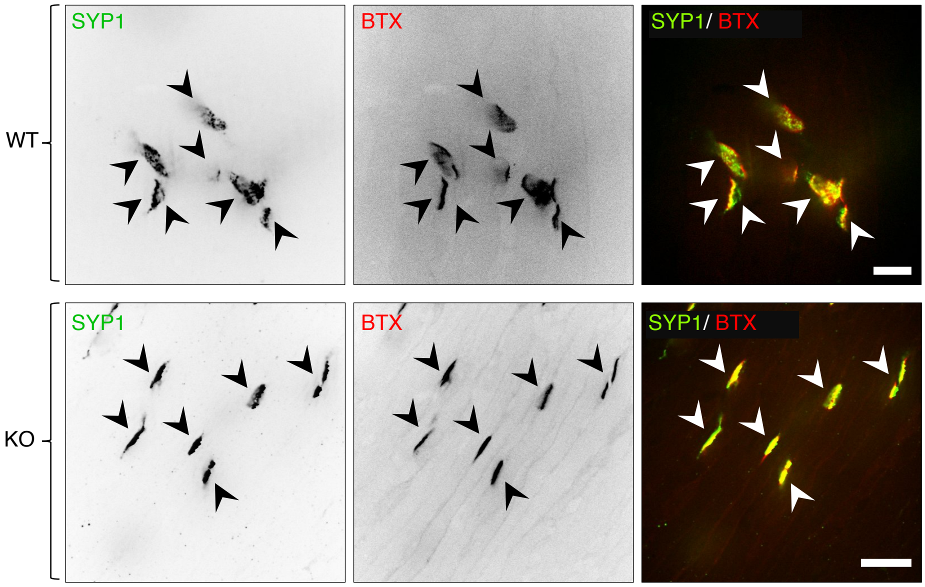

Supplemental Information

Figure S1. Distinct Transport of CD63-GFP and SYG1-Halo in the Axon of Rat Hippocampal Neurons. Related to Figure 1.

DIV4 rat hippocampal neurons were co-transfected with plasmids encoding CD63-GFP and SYG1-Halo. At DIV5, vesicle movement in the axon was analyzed as described in the legend to Figure 1. Single-color images are shown in inverted grayscale. In the merge panel, green (CD63-GFP) and red (SYG1-Halo) and lines represent moving vesicles having one or the other marker, and blue lines represent vesicles having both markers. Quantification of 54 vesicles in 5 axons showed that 22.2 ± 7.8% of SYG1-Halo-containing vesicles also contained CD63-GFP.

Figure S2. Depletion of Axonal Lysosomes by RAMP Does Not Affect the Localization of VMAT2 to the Axon. Related to Figure 2.

(A) DIV5 rat hippocampal neurons were co-transfected with plasmids encoding LAMP1-SBP-RFP and Strep-KIFC1*-HA, together with VMAT2-GFP. Neurons were cultured in the presence of NeutrAvidin to remove biotin from the medium. After 24 h, neurons were fixed and immunostained for HA and endogenous ankyrin G (ANKG), and examined by confocal fluorescence microscopy. Bars: 20 μm. Arrowheads indicate the axon. (B) Straightened and enlarged 50-μm segments of axons from the box in panel A showing the depletion of LAMP1-SBP-RFP, but not VMAT2-GFP, from the axon of neurons expressing Strep-KIFC1*-HA in the absence of biotin.

Figure S3. ARL8B Co-Moves with Lysosomes but not SVPs. Related to Figure 1.

(A) DIV4 rat hippocampal neurons were co-transfected with plasmids encoding ARL8B-mCherry with LAMP1-GFP (top row) or SYG1-GFP (bottom row). At DIV5, vesicle movement in the distal axon was analyzed as described in the legend to Figure 1. Single-color images were represented in inverted grayscale. In the merge panel, red (ARL8B-mCh) and green (LAMP1-GFP or SYG1-GFP) lines represent moving vesicles having one or the other marker, and blue lines represent vesicles having both markers. (B) Quantification of the number of anterograde-moving LAMP1-GFP or SYP1-GFP vesicles that were also positive for ARL8B-mCh. Values are the mean ± SD of >100 vesicles in 7 neurons per condition, and are expressed as the percentage of the indicated marker that co-moves with ARL8B-mCh in the distal axon. Significance is denoted by asterisks: ***$p < 0.005$ as per Student's t test.

Figure S4. Sequence Alignment of WT and Mutant Myrlysin. Related to Figure 3A.

Deletion of 10 bp from exon 2 is predicted to cause a frameshift after K31 and the addition of the sequence SWW at the C-terminus of the truncated protein. The asterisk indicates a stop codon. Amino-acid numbers are indicated on top and on the right.

Figure S5. Analysis of the distribution of SV and Lysosomal proteins in Rat and Mouse Neurons. Related to Figure 5, 6 and 7A.

(A,B) Straightened and enlarged 20-μm segments of axons from DIV6 rat hippocampal neurons stained for endogenous LAMTOR4 and SV2 (A) or DIV14 rat hippocampal neurons co-transfected with plasmids encoding SYP1-mCherry and CD63-GFP for 24 h (B). Axons were examined by confocal microscopy (A) or by structure illumination microscopy (B). Top rows show single-channel images in inverted grayscale and merged images in color. Line intensity scans are shown for lysosomal and SV proteins. Notice the overlapping but distinct localization of lysosomal and SV proteins in the axons. (C) DIV12 cultures of hippocampal neurons from WT and myrlysin-KO E18 mouse embryos were immunostained for endogenous SYP1 together with post-synaptic density protein 95 (PSD-95) and microtubule associated protein 2 (MAP2, dendrite marker). Images on the left three columns show single-channel images in inverted grayscale and images on the right-most column show merged images. Magnified views of the boxed areas are shown the second and fourth rows. Bars: 20 μm. The co-localization of SYP1 with PSD-95 in both WT and myrlysin-KO neurons indicate that myrlysin is not required for accumulation of SYP1 at synapses.

Figure S6. Normal Staining for Synaptophysin 1 at Diaphragm Neuromuscular Junctions of Myrlysin-KO Mice. Related to Figure 7A.

Whole diaphragms were harvested from WT and myrlysin-KO mouse E18 littermates and co-stained with antibody to SYP1 and Alexa Fluor 594-conjugated α-Bungarotoxin (BTX). Single-channel images are shown in inverted grayscale and merged images are shown in color. Arrowheads point to individual NMJs. Bars: 20 μm. Notice the presence of SYP1 at NMJs from both WT and KO mice.

Table S1. Velocity of Anterograde Vesicles in the Axon of Rat Hippocampal Neurons. Related to Figure 1B.

Construct:	Axon region	Mean velocity (μm/s)	SD
LAMP1-GFP	Proximal	1.5	0.3
LAMP1-GFP	Distal	1.7	0.6
SYG1-GFP	Proximal	2.0	0.6
SYG1-GFP	Distal	1.9	0.6
SYP1-YFP	Proximal	2.3	0.6
SYP1-YFP	Distal	2.2	0.8
VMAT2-GFP	Proximal	1.8	0.6
VMAT2-GFP	Distal	2.0	0.5

Anterograde movement of LAMP1-GFP, SYG1-GFP, SYP1-YFP and VMAT2-GFP vesicles in the proximal and distal axon. Values are the mean ± SD of >100 vesicles in 6 neurons per condition, and were calculated using Fiji from kymographs such as those shown in Figure 1B.
